# Biological evaluation of novel gemcitabine analog in patient-derived xenograft models of pancreatic cancer

**DOI:** 10.1186/s12885-023-10928-w

**Published:** 2023-05-13

**Authors:** Andriana Inkoom, Nkafu Bechem Ndemazie, Taylor Smith, Esther Frimpong, Raviteja Bulusu, Rosemary Poku, Xue Zhu, Bo Han, Jose Trevino, Edward Agyare

**Affiliations:** 1 College of Pharmacy and Pharmaceutical Sciences, Florida A&M University, 1415 South Martin Luther King Jr Blvd, Tallahassee, FL 32307 USA; 2 College of Medicine, Central Michigan University, Mount Pleasant, MI 48859 USA; 3 Department of Surgery, Keck School of Medicine University of Southern California, Los Angeles, California 90033 USA; 4 Department of Surgery, University of Florida College of Medicine, Gainesville, FL 32610 USA; 5 Department of Surgery, College of Medicine, Virginia Commonwealth University, Richmond, VA 23298 USA

**Keywords:** Black, White, 4-N-stearoyl Gemcitabine, Solid-lipid nanoparticle, Pancreatic cancer, Antitumor efficacy, Patient-derived xenograft model

## Abstract

Gemcitabine (Gem) has been a standard first-line drug for pancreatic cancer (PCa) treatment; however, Gem's rapid metabolism and systemic instability (short half-life) limit its clinical outcome. The objective of this study was to modify Gem into a more stable form called 4-(N)-stearoyl-gemcitabine (4NSG) and evaluate its therapeutic efficacy in patient-derived xenograft (PDX) models from PCa of Black and White patients.

**Methods **4NSG was synthesized and characterized using nuclear magnetic resonance (NMR), elemental analysis, and high-performance liquid chromatography (HPLC). 4NSG-loaded solid lipid nanoparticles (4NSG-SLN) were developed using the cold homogenization technique and characterized. Patient-derived pancreatic cancer cell lines labeled Black (PPCL-192, PPCL-135) and White (PPCL-46, PPCL-68) were used to assess the in vitro anticancer activity of 4NSG-SLN. Pharmacokinetics (PK) and tumor efficacy studies were conducted using PDX mouse models bearing tumors from Black and White PCa patients.

**Results **4NSG was significantly stable in liver microsomal solution. The effective mean particle size (hydrodynamic diameter) of 4NSG-SLN was 82 ± 6.7 nm, and the half maximal inhibitory concentration (IC50) values of 4NSG-SLN treated PPCL-192 cells (9 ± 1.1 µM); PPCL-135 (11 ± 1.3 µM); PPCL-46 (12 ± 2.1) and PPCL-68 equaled to 22 ± 2.6 were found to be significantly lower compared to Gem treated PPCL-192 (57 ± 1.5 µM); PPCL-135 (56 ± 1.5 µM); PPCL-46 (56 ± 1.8 µM) and PPCL-68 (57 ± 2.4 µM) cells. The area under the curve (AUC), half-life, and pharmacokinetic clearance parameters for 4NSG-SLN were 3–fourfold higher than that of GemHCl. For in-vivo studies, 4NSG-SLN exhibited a two-fold decrease in tumor growth compared with GemHCl in PDX mice bearing Black and White PCa tumors.

**Conclusion **4NSG-SLN significantly improved the Gem's pharmacokinetic profile, enhanced Gem's systemic stability increased its antitumor efficacy in PCa PDX mice bearing Black and White patient tumors.

**Supplementary Information:**

The online version contains supplementary material available at 10.1186/s12885-023-10928-w.

## Introduction

Pancreatic cancer (PCa) is a global problem and has emerged as one of the highly fatal types of malignancies in the United States of America. It has an annual incidence of more than 62,210 cases (32,970 men and 29,240 women) and a yearly mortality rate of almost 49,830 cases (25,970 men and 23,860 women) and has the worst 5-year survival rate of all cancers [1, 2]. About 90% of PCa is characterized as pancreatic ductal adenocarcinoma (PDAC) with desmoplastic stroma, which prevents the efficient delivery of chemotherapeutic drugs [3]. Despite the advancement in PCa research and treatments, the Surveillance, Epidemiology, and End Results (SEER) program report still indicates a higher incidence in Blacks followed by Whites. Aside from socioeconomic status and lifestyle, the primary reason for the high mortality in these populations is attributed to the absence of screening tests to detect PCa in patients at the early stages with little to no symptoms [4, 5].

Also, rapid growth, early dissemination to distant organs, and resistance to chemotherapy contribute to the low survival records in PCa patients [6, 7]. Tyrosine kinase receptors such as epidermal growth factor receptor (EGFR), ErbB2/human epidermal growth factor receptor 2 (HER2), and vascular endothelial growth factor receptor (VEGFR) are highly expressed in several solid tumors, including PCa [8, 9]. Overexpression and mutation of these receptors may result in proliferation, migration, and differentiation required for PCa pathogenesis [10]. These receptors have been shown to confer poor treatment outcomes in pancreatic cancer [11, 12].

Current treatment of PCa using chemotherapy is primarily based on nucleoside analogs. These molecules are designed to mimic natural pyrimidine, purine nucleosides, and gemcitabine (Gem), one of the analogs [13].

As monotherapy, Gem is currently the preferred first-line therapeutic agent for advanced metastatic PCa. Modified FOLFIRINOX (mFOLFIRINOX), a combination therapy comprising of (5-fluorouracil (5FU), leucovorin, oxaliplatin, and irinotecan), albumin-bound paclitaxel ((Nab-PTX, Abraxane®) together with Gem are generally considered the optimal adjuvant chemotherapeutic regimen for PCa. Gem has improved the quality of life and increased the survival rate of patients with unresectable PCa [14]. However, the systemic instability of Gem (plasma circulation half-life < 15 min) has rendered it less effective due to its rapid metabolism to an inactive metabolite (2′,2′- difluorouridine) by cytidine deaminase. Gem is administered at a high dose (1000 mg/m^2^) to increase its therapeutic levels, resulting in serious side effects such as renal and hematological toxicities [15, 16],. Therefore, Gem needs to improve its systemic stability and bioavailability and prolong its half-life.

Nanotechnology and its applications in drug therapy have become the fastest-growing area adopted by formulation experts to overcome challenges associated with the delivery of lipophilic or metabolic unstable drugs. Novel nano-delivery systems such as liposomes, micelles, polymeric nanoparticles, and dendrimers have recorded tremendous progress in their applications in drug delivery. Among these delivery systems, lipid-based nanocarriers are the most popular and preferred because they are highly biocompatible and biodegradable [17]. Solid lipid nanoparticle (SLN) has proven to be a novel drug delivery system for hydrophilic and hydrophobic drugs [18, 19]. SLNs enhance the physicochemical properties of nanoparticles, extravasate into tumors via their leaky vasculature, and increase localized tumor drug exposure based on the enhanced permeation and retention (EPR) effect [20].

Commercially available PCa cell lines are used frequently in animal models for drug screening and to assess the sensitivity of drugs to tumors. Nonetheless, these cells have poor predictive and translational value due to the high passages established over the past 40 years [21]. In addition, commercially established PCa cells have less significant molecular heterogeneity, which provides a limited comprehensive understanding of patients' responses to chemotherapy. This necessitates using patient-derived cells and xenograft mouse models to assess the efficacy of chemotherapeutic agents against pancreatic cancer. Patient-derived cells have been proven to be highly predictive of clinical outcomes for PCa treatment and have translational value compared to commercially available PCa cell lines.

Previously, we synthesized 4NSG from Gem and stearic acid and tested its anticancer efficacy against PCa cell lines. 4NSG remarkably reduced tumor growth in pancreatic patient-derived xenograft (PDX) mouse model with no morphological changes to mice's liver and kidney tissues [22].

This study focuses on developing and characterizing 4NSG-SLN and testing its efficacy against patient-derived pancreatic cancer cells (PPCLs) in vitro from Black (PPCL-192, PPCL-135) and White (PPCL-46 and PPCL-68) patients as well as in PDX mouse models with tumors from Black and White patients.

**Fig. 1:**
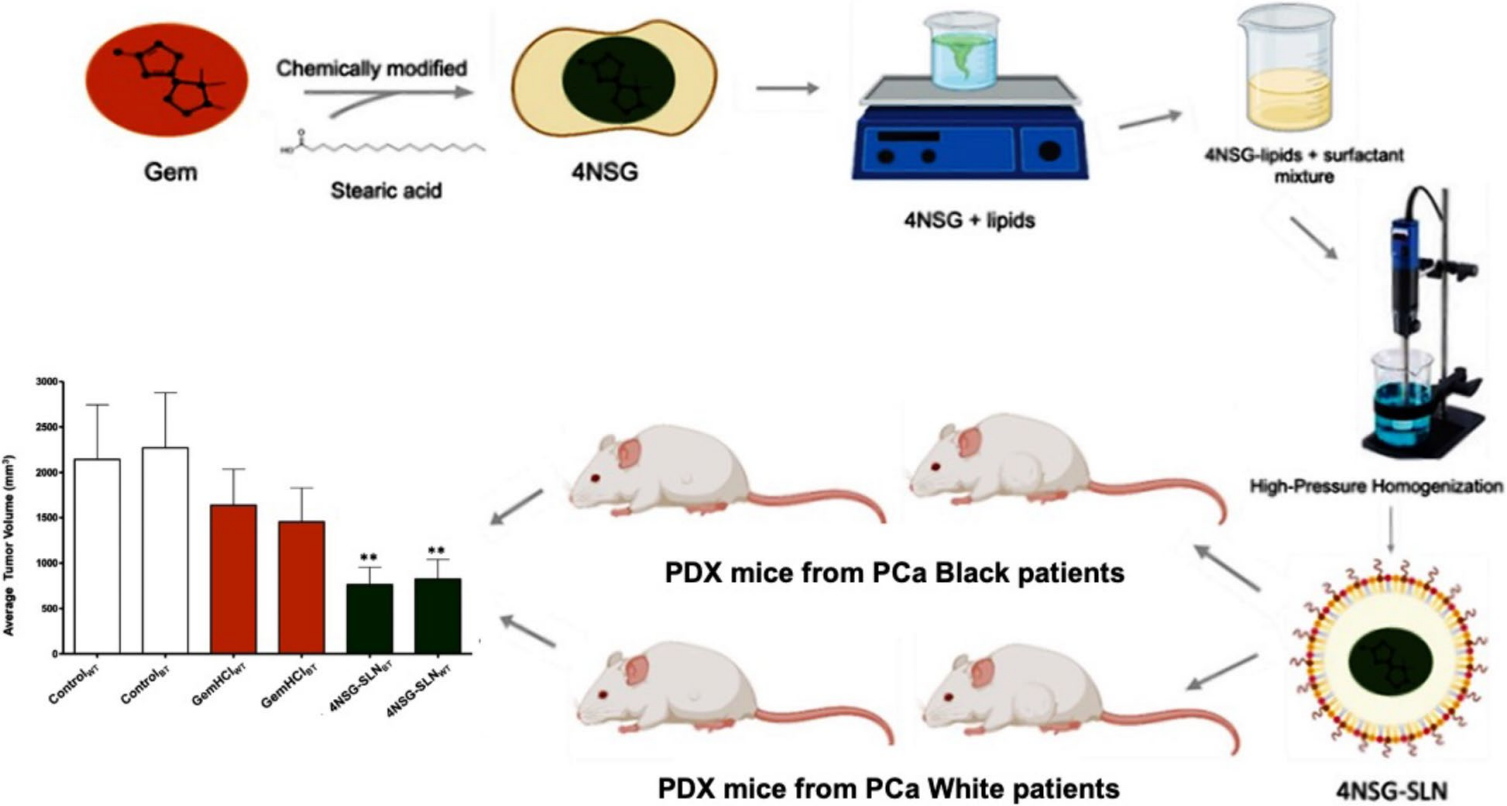
Graphical summary of novel gemcitabine analog evaluation in pancreatic cancer patient-derived xenograft models

We conducted cytotoxicity and cellular uptake of 4NSG-SLN against PPCL-192, PPCL-135, PPCL-46, and PPCL-68 cells. In this study, using 4NSG-SLN, we demonstrated systemic stability of Gem, significant tumor growth inhibition in PDX mouse models in mice with Black and White PCa tumors, and the enhanced pharmacokinetic profile and improved therapeutic efficacy of Gem. This work demonstrated the first time Gem analog has been investigated in PDX mouse models bearing tumors from Black and White pancreatic cancer patients (Fig. 1).

## Materials and methods

Gemcitabine hydrochloride (GemHCl) was purchased from AK Scientific (Union City, CA). Caprylocaproyl polyoxyl-8 glycerides (Labrasol) and Soy lecithin were obtained from Millipore Sigma Aldrich (Burlington, MA). Patient-derived primary pancreatic cancer cells from Black (PPCL-192 and PPCL-135) and White (PPCL-46 and PPCL-68) patients were obtained from Dr. Trevino's laboratory with methods previously described [21]. All other materials, solvents, and analytical-grade reagents were purchased from Sigma Aldrich (Louis, MO).

### Synthesis of 4NSG

**Fig. 2:**
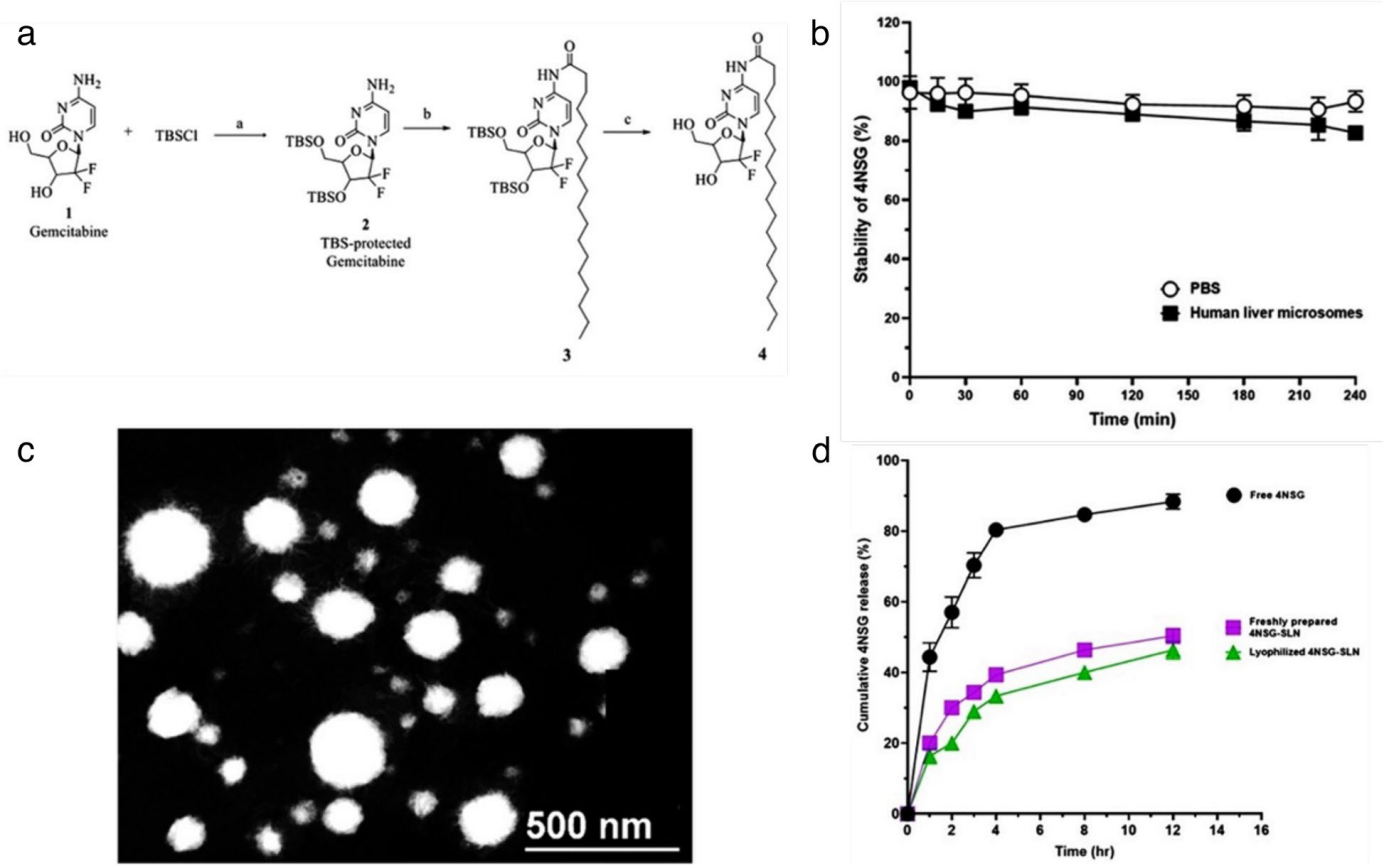
Synthesis, characterization, and in vitro release of 4NSG: (**a**) Reagents and conditions for the synthesis of 4NSG (4): a) TBSCl, imidazole, DCM, rt b) DIPEA, stearic anhydride, DCM, 24 h c) TBAF DMF, rt, 2 h**,** (**b**) In vitro metabolic stability of 4NSG in PBS and human liver microsome solution (Data expressed as mean ± SD, *n* = 3), (**c**) Transmission Electron Micrograph (TEM) micrograph of 4NSG-SLN (Scale bar = 500 nm), (**d**) Cumulative 4NSG release from free 4NSG, freshly prepared 4NSG-SLN, and lyophilized 4NSG-SLN in PBS (phosphate-buffered saline; pH 7.4 at 37 °C)

The 4NSG was synthesized following a method described by Trung et al. [23, 24] with slight modifications. Gemcitabine **1** (2.630 g, 9.992 mmol, 1 eq) was dissolved in 100 mL dichloromethane (50.0 mL). To the solution was added *tert*-butyldimethylsilyl (TBS) chloride (3.765 g, 24.981 mmol, 2.5 eq) and imidazole (2.041 g, 29.977 mmol, three eq) successively. The reaction mixture was stirred at room temperature until Gem was consumed, as indicated by thin-layer chromatography (TLC) analysis (using 100% ethyl acetate (EtOAc)). The mixture was washed with saturated ammonium chloride, sodium bicarbonate, and sodium chloride solutions. The organic layer was dried with anhydrous sodium sulfate, filtered, and concentrated under a vacuum. The TBS-protected compound **2** was crystallized from EtOAc as a white solid and used in the next step without further purification (Fig. 2a) [23].

Next, to a solution of N, N-diisopropylethylamine (DIPEA) (1.200 eq) in dichloromethane (DCM) was added stearate anhydride (1.100 eq)-warmed to dissolve, and TBS-protected gemcitabine **2** (~ 1.000 eq). The resulting mixture was stirred for 24 h at room temperature. After, the excess solvent was removed under reduced pressure. The residue containing intermediate **3** obtained was used in the next step without further purification.

A mixture of 3 (1 g, 1.4 mmol) and tetra-n-butylammonium fluoride (TBAF) in dimethylformamide (DMF) (15 mL) was stirred at room temperature for 2 h. Excess solvent was removed, and the resulting residue was directly purified on silica-gel flash column chromatography with gradient DCM-ethanol (up to 15% ethanol) to afford 4NSG (4) as a white solid [23].

The 4NSG was synthesized according to Fig. 2a and analyzed by proton and carbon-13 NMR, elemental analysis, HPLC, MS, and TLC. The melting point of the solid 4NSG analog was determined. The purity of 4NSG was greater than 99.6%, determined by HPLC and elemental analysis. Elemental analysis was conducted by Atlantic Microlab, Inc, Norcross, GA.

### 4NSG

^1^H NMR (600 MHz, DMSO-d6) δ 10.93 (s, 1H), 8.20 (d, J = 7.6 Hz, 1H), 7.25 (d, J = 7.7 Hz, 1H), 6.27 (d, J = 6.5 Hz, 1H), 6.14 (t, J = 7.4 Hz, 1H), 5.27 – 5.24 (m, 1H), 4.19 – 4.14 (m, 1H), 3.86 (d, J = 8.4 Hz, 1H), 3.80 – 3.75 (m, 1H), 3.65 – 3.59 (m, 1H), 2.40 – 2.34 (m, 2H), 1.51 (t, J = 7.4 Hz, 2H), 1.26 – 1.15 (m, 28H), 0.82 (t, J = 7.0 Hz, 3H).

^13^C NMR (151 MHz, DMSO) δ 174.53, 163.33, 154.64, 145.16, 123.38, 96.34, 84.55, 81.47, 68.83, 59.23, 36.83, 31.72, 29.46, 29.44, 29.42, 29.40, 29.27, 29.13, 28.87, 24.78, 22.52, 14.38. MP = (145–146 °C), Rf value = 0.54 (100% ethyl-acetate). HPLC (H_2_O/ACN (90:10), % purity = 99.8%, RT = 14.3 min) MS (M + H)^+^  = 530.59, Molecular formula: C_27_H_45_F_2_N_3_O_5_•H_2_O, Mol wt: (4NSG) 547.68. Elemental Analysis: Calculated: C = 59.21; H = 8.65; *N* = 7.67; F = 6.94. Found: C = 59.27; H = 8.59; *N* = 7.51; F = 6.67.

### In vitro metabolic stability of 4NSG

The metabolic stability of 4NSG was examined in the presence of PBS (pH 7.4) and human liver microsomes as previously described [25, 26]. Human liver microsomes were obtained from Sigma-Aldrich. 4NSG (10 µM) was spiked into PBS, or liver microsomes (1 mg protein/mL) and incubated in a shaking water bath at 37 °C for 8 h. Aliquots (100 µL) were taken after 0, 15, 30, 60, 120, 180, 220 and 240 min of incubation and immediately pretreated with an ice-cold methanolic solution including the internal standard, vortexed for 3 min and centrifuged (12,000 × g, 10 min). Supernatants were stored at -80 C until required for analysis [25, 26].

### HPLC analysis of 4NSG

To determine concentrations of 4NSG in PBS and human liver microsomes solutions, supernatants from PBS and human liver microsomes controls were spiked with aliquots of 0.5 μg/ml of 4NSG. The chromatographic analysis was performed according to the method described with minor modifications [27, 28]. The samples were prepared using aliquots of supernatants (10µL) of control (spiked), PBS, and human liver microsomes. The chromatographic system consisted of an HPLC (Agilent Technologies, 1290 infinity) equipped with an auto-sampler, diode array detector (DAD), and pumps. Separation was performed using a reverse phase column (Zobrax Rapid Resolution High Definition (RRHD) Eclipse plus C18, 2.1 × 50 mm, 1.8 µm). A flow rate of 250 µL/min and injection volume of 10 μL at ambient temperature was maintained at 25 ± 1 °C while detection was performed at 268 nm. Before analysis, the reverse phase column was equilibrated with a mobile phase of 5% acetonitrile in 10 mM dihydrogen phosphate buffer, pH adjusted to 2.5 with trifluoroacetic acid (TFA). Isocratic elution was performed throughout the entire analysis, including internal standards. A calibration curve was prepared using 4NSG standard solutions with a concentration range of 0.063–2.0 μg/mL. A plot of the peak areas as a function of 4NSG concentration was plotted, and the linear equation of the calibration curve given as y = mx + c was determined, where y is the peak area, m is the slope, x is the concentration of 4NSG, and c is the y—intercept.

### Preparation of 4NSG-SLN

4NSG-SLN formulation was prepared by cold homogenization technique using a high-shear homogenizer technique with slight modifications [29]. Briefly, 30 mg of 4NSG was added to 3 mL of labrasol in a glass vial and was heated to 70–80 °C to melt. The 4NSG-containing lipid melt was cooled to produce a solid lipid ground to lipid microparticles (approximately 50-100 μm), followed by the dispersion in a 10 mg/mL cold lecithin solution and further stabilized with Tween 80 (1% w/v ratio) to produce a pre-suspension. The yielded pre-suspension was homogenized (1,200 rpm (3,820 rcf), 3–5 cycles) on an ice bath (below room temperature) and directly broken down into solid lipid nanoparticles. Freshly prepared SLN formulations were characterized and used for in vitro and in vivo studies. The 4NSG-SLN formulation was lyophilized with 5% w/v mannitol and stored at 4 °C for future use.

### Characterization of 4NSG-SLN formulation

#### Transmission electron microscopy (TEM)

The morphological examination of 4NSG-SLN was performed using high-resolution TEM. Three microliters (3 mL) sample was stained using 50 μL of ammonium molybdate solution (1% w/v) after adjusting the pH of the 4NSG-SLN suspension to 7.0 with 5 N sodium hydroxide. The stained sample was placed on copper grids, allowed to dry, and viewed by a Tecnai F-20 transmission electron microscope (Philips Co. Japan).

### Particle size and zeta potential

The particle size, polydispersity (PDI), and zeta potential were measured using a Zeta Potential/Particle Sizer (NICOMP 380 ZLS). All measurements were performed in triplicate. The NICOMP 380 ZLS estimated the nanoparticles based on principles of dynamic light scattering (DLS).

### In vitro stability of 4NSG-SLN

The stability of 4NSG-SLN was analyzed after incubation in PBS (pH = 7.4) alone (control) and PBS with 25% (v/v) FBS. Both solutions were maintained at 37 °C in a water bath for 7 days. NICOMP 380 ZLS particle sizer was used to measure the hydrodynamic diameter and PDI of 4NSG-SLN in both PBS alone and PBS with 25% (v/v) FBS for 7 consecutive days.

### Entrapment Efficiency (EE) of 4NSG in SLN

The entrapment efficiency of 4NSG-SLN was determined by suspending 10 mg of lyophilized 4NSG-SLN in 2 mL of PBS (pH 7.4). The mixture was centrifuged at 6000 rpm for 5 min, and the supernatant was subjected to HPLC (Waters Corporation, Milford, MA) equipped with an auto-sampler photodiode array (2998 UV/Vis) detector and pumps. Separation was performed using a reverse-phase column (ZORBEX SB – C18 4.6 × 250 mm, 5 μm) with a flow rate of 1.0 ml/min and injection volume of 20 μL at ambient temperature at 268 nm. The EE was calculated based on the equation below:

1 $$\boldsymbol E\boldsymbol E\boldsymbol=\frac{\mathbf W\mathbf e\mathbf i\mathbf g\mathbf h\mathbf t\boldsymbol\;\mathbf o\mathbf f\boldsymbol\;\mathbf4\mathbf N\mathbf S\mathbf G\boldsymbol\;\mathbf e\mathbf n\mathbf t\mathbf r\mathbf a\mathbf p\mathbf p\mathbf e\mathbf d\boldsymbol\;\mathbf i\mathbf n\boldsymbol\;\mathbf n\mathbf a\mathbf n\mathbf{op}\mathbf a\mathbf r\mathbf t\mathbf i\mathbf c\mathbf l\mathbf e\mathbf s}{\mathbf I\mathbf n\mathbf i\mathbf t\mathbf i\mathbf a\mathbf l\boldsymbol\;\mathbf w\mathbf e\mathbf i\mathbf g\mathbf h\mathbf t\boldsymbol\;\mathbf o\mathbf f\boldsymbol\;\mathbf4\mathbf N\mathbf S\mathbf G\boldsymbol\;\mathbf u\mathbf s\mathbf e\mathbf d}\mathbf x\mathbf\;\boldsymbol{100}$$

  


### In vitro release of 4NSG from SLN

Cumulative in vitro drug release studies were performed as previously described [30, 31]. Free 4NSG (3 mg), freshly prepared and lyophilized 4NSG-SLNs containing approximately 5 mg (suspended in 2 mL PBS) were placed in dialysis bags with molecular weight cut-off (MWCO) of 3.5 kDa) and immersed in their respective labeled 15 mL PBS, (pH 7.4) as the release medium and incubated at 37 °C. The free 4NSG, freshly prepared and lyophilized 4NSG-SLNs solutions were gently stirred at 100 rpm 1 mL aliquots were withdrawn and replenished with fresh PBS (maintained at 37 °C) at 0, 1, 2, 4, 8, and 12 h for analysis. The samples were analyzed for the presence of 4NSG using the HPLC system previously described under the HPLC analysis of 4NSG of the in vitro metabolic stability of the 4NSG section.

### Cell viability studies

PCa primary cell were cultured with Dulbecco's modified Eagle medium (DMEM) with high glucose and L-glutamine and supplemented with 10% fetal bovine serum (FBS) and 1% penicillin–streptomycin (PenStrep) [32]. Briefly, PPCL-192, PPCL-135, PPCL-46, and PPCL-68 were seeded at a density of 1 × 10^3^ per well in 96-well plates in triplicates for each drug concentration level and incubated at 5% CO_2_ and a temperature of 37 °C [32]. At 70 -75% confluency, PPCL-192, PPCL-135, PPCL-46, and PPCL-68 cells were treated with GemHCl, 4NSG-free SLN, and 4NSG-SLN. Varying concentrations of 4NSG-SLN and 4NSG-free SLN were prepared by diluting their stock solutions with a growth medium. For GemHCl, a stock solution was prepared with phosphate-buffered saline (PBS) and serially diluted with a growth medium to prepare different concentrations: 3, 6, 12, 25, 50, and 100 µM. The cells were treated with 100 µL of each drug concentration in triplicates and incubated for 48 h. At termination, 20 µL of 0.05% resazurin sodium salt (Alamar blue®) was added and incubated at optimum conditions (5% CO_2_, 37 °C) for 2–4 h [33]. GloMax® Explorer Multimode Microplate reader with a fluorescence excitation wavelength of 560/580 nm and emission wavelength of 590/610 nm was used to determine the percent viable cells per concentration calculated.

### Cellular uptake

#### Confocal imaging

PPCL-192 and PPCL-46 cells were grown in 6-well plates (with coverslips) at a cell density of 2 × 10^3^ for 24 h at 37 °C. The cells were treated with fluorescein isothiocyanate (FITC)-labeled SLN in the growth medium. After 3 h, FITC-SLN was removed, and the cells were gently washed twice with PBS. Afterward, 5 μg/ml of Hoechst dye was added for nuclear staining; the cells were fixed using 4% paraformaldehyde. The fixed cells were mounted and imaged using Leica SP2 Multiphoton system [34].

### Flow cytometry

PPCL-192 and PPCL-46 cells were seeded at a cell density of 1 × 10^5^ in 6-well plates and cultured in a growth medium until 70% confluence to determine cellular uptake of 4NSG-SLN. In place of 4NSG, cells were treated with FITC and (FITC)-labeled SLN for 24 h at 37 °C. After treatment, cells were washed thrice with PBS and detached using 0.25% trypsin- ethylene diamine tetraacetic acid (EDTA) solution. Trypsin was deactivated by adding a culture medium and centrifuged at 6,000 rpm (3820 rcf) for 5 min. Cells were resuspended in 500 μl PBS and fixed with 4% paraformaldehyde. The fixed cells were kept on ice until analysis using a Becton Dickinson (BD) Fluorescence-Activated Cell Sorting Canto Analyzer and a BD Fluorescence-Activated Cell Sorting Aria Cell Sorter (BD Biosciences) [34].

### Colony formation assay

For colony assay, PPCL-192 and PPCL-46 cells were seeded into a T-25cm^2^ culture flask at a density of 5 × 10^5^ cells and cultured in DMEM medium supplemented with 2 mM L-glutamine, 10 mM HEPES, 10% FBS, and 1% penicillin/streptomycin. After the cells reached 75% confluency, they were exposed to varying concentrations of (5 µM, 10 µM, and 20 µM) for GemHCl and 4NSG-SLN. After 48 h exposure, treatment was terminated, cells harvested, and then re-plated onto 6-well plates at a density of 200, 500, and 1,000 cells per well and incubated with a growth medium. After the cells reached 75% confluence, the experiment was terminated by fixing and staining the plates with 0.5% crystal violet solution. The stained colonies (fifty per colony) were counted using a Jenco™ Stereomicroscope; plating efficiency (PE) and surviving fraction (SF) were calculated, and a graph of survival curve graph was generated [35].

### Cell cycle studies

Cell cycle study was performed to determine the effect of 4NSG-SLN on the cell cycle; PPCL-192 and PPCL-46 cells (2 × 10^5^ cells per well) were seeded in 6-well plates and cultured using DMEM medium supplemented with 10% FBS10 mM HEPES, 10% FBS, and 1% penicillin/streptomycin were seeded in 6-well plates and incubated at 37 °C. After the cells reached 75% confluence, they were exposed to different concentrations (5 μM,10 μM, and 20 µM) of GemHCl and 4NSG-SLN. After 24 h exposure, cells were harvested, washed with PBS, centrifuged at 300 × g for 5 min, resuspended the pellets in 100 μL of PBS, and passed through a 28 5/8 needle to disperse them into single cells. Fixation and permeation of cells were performed using cold 70% ethanol while vortexing. Cells were then stored at − 20 °C overnight. The cells were resuspended in 0.1 mg/mL RNase, stained with 40 mg/mL PI, and the phase distribution was examined using flow cytometry (FACScalibur-Becton Dickinson).

### Cell migration

A cell migration assay was conducted to determine the effect of 4NSG-SLN on PPCL-192 and PPCL-46 cell motility. The Ibidi cell culture inserts were used to grow the cells into two confluent monolayers separated by a "wound" for this assay. The cells were seeded into 24-well plates at a cell density of 2.5 × 10^5^, 48 h at 37 °C. At 75% confluency, cells formed adherent monolayers on either side of the tissue culture insert. Before treatment, the cells were serum-starved by replacing complete media with base media (DMEM) and then further incubated for 48 h. After serum starvation, the inserts were gently removed to generate a gap or "wound" between the two confluent layers of cells, and the monolayers were washed with experimental media. Afterward, cells were exposed to 5 μM concentration of GemHCl and 4NSG-SLN treatments. Images of the cells invading the wound were then captured after 48 h with a Nikon Ti Eclipse microscope.

### Animal studies

Eight-week-old mice were obtained from The Jackson Laboratory (Bar Harbor, ME).

#### Ethics statements

The mice were housed in a virus-free, indoor, light- and temperature-controlled barrier environment and were provided ad libitum access to food and water. All procedures with mice were in strict accordance with the National Institutes of Health Guide for the Care and Use of Laboratory Animals and the Animal Research Reporting of In Vivo Experiments (ARRIVE) guidelines.This was approved by the Florida A & M University Animal Care and Use Committee**.**

#### Tumor transplantation

The implantation of surgical tumor tissue into immuno-compromised mice was described previously [21, 36]. A viable portion of resected tissue 2 × 2 mm in size was briefly isolated immediately from surgically resected primary PCa specimens (from Blacks and White patients) with care to minimize critical ischemia time. The PCa tissues were then implanted subcutaneously into an 8-week-old mouse (The Jackson Laboratory, Bar Harbor, ME). The xenografts were allowed to grow to a maximum diameter of 1.5 cm before passage and in vitro culture.

### Pharmacokinetic studies

For pharmacokinetic (PK) studies, normal healthy mice were grouped into control, GemHCl, and 4NSG-SLN. Mice were given a single bolus intravenous injection of 30 mg/kg of Gem (with Gem equivalent dose of GemHCl and 4NSG-SLN), while control mice received normal saline (0.9% NaCl). After the injection, aliquots of blood samples were collected at predetermined time points (5, 10, 15, 30, 60, 120, 240, 360, 720, and 1440 min). Blood samples collected were treated with 2 mL of an extraction solvent (15% of isopropyl alcohol in ethyl acetate) [37, 38]. The mixture was vortexed for 30 s and centrifuged at 3,000 rpm for 15 min, and the supernatant was collected and vaporized to dry overnight in a water bath. Residual solvent was removed by placing the dried samples in vacuum chamber [22].

Five hundred microliters (500 uL) of mobile phase solution was used to reconstitute the dried sample (mobile phase solution was made up of 5% acetonitrile in 10 mM dihydrogen phosphate buffer, pH adjusted to 3). The sample solution was centrifuged at 3,000 rpm for 15 min, supernatant collected and filtered, and samples analyzed for Gem using HPLC. The PK parameters were determined using PKSolver software [39].

#### HPLC analysis of Gem

To determine the concentration of Gem, supernatant from mouse plasma was spiked with aliquots of 0.5 μg/ml of Gem. The chromatographic analysis was performed according to the method described with minor modifications [27, 28]. The samples were collected using supernatants (10µL), aliquots of control (spiked), and mouse plasma. The chromatographic system consisted of an HPLC (Agilent Technologies, 1290 infinity) equipped with an auto-sampler, diode array detector (DAD), and pumps. Separation was performed using a reverse phase column (Zobrax Rapid Resolution High Definition (RRHD) Eclipse plus C18, 2.1 × 50 mm, 1.8 µm). A flow rate of 250 µL/min and injection volume of 10 μL at ambient temperature was maintained at 25 ± 1 °C while detection was performed at 268 nm. Before analysis, the reverse phase column was equilibrated with a mobile phase of 5% acetonitrile in 10 mM dihydrogen phosphate buffer, pH adjusted to 2.5 with trifluoroacetic acid (TFA). Isocratic elution was performed throughout the entire analysis, including internal standards. A calibration curve was prepared using Gem standard solutions with a concentration range of 0.063–2.0 μg/mL. A plot of the peak areas as a function of Gem concentration was plotted, and the linear equation of the calibration curve given as y = mx + c was determined, where y is the peak area, m is the slope, x is the concentration of Gem, and c is the y—intercept.

### Immunohistochemistry

Tissue samples from mice bearing tumors from Black and White PCa patients post treatments with GemHCl and 4NSG-SLN were excised and immediately washed with PBS, fixed (10% buffered formalin) for 24 h, and transferred to 70% ethanol for histopathological analysis. Histology was performed by HistoWiz Inc. (Histowiz.com) according to manufacturer protocol. Samples were embedded in paraffin, and 4 μm sections were prepared [7, 40, 41]. Immunohistochemistry was performed on a Bond Rx autostainer (Leica Biosystems) with enzyme treatment (1:1000) using standard protocols (https://home.histowiz.com/faq/). Samples were incubated overnight with primary antibodies (Antibodies used were rat monoclonal F4/80 primary antibody (eBioscience, 14–4801, and 1:200), and immunohistochemical staining was done using rabbit anti-rat secondary (Vector Labs, 1:100) [7, 40, 41]. Bond Polymer Refine Detection was used per the manufacturer's protocol (Leica Biosystems). After staining, sections were dehydrated and filmed, coverslipped using a Tissue-Tek Prisma coverslip (Sakura). Whole slide scanning (40x) was performed on an Aperio AT2 (Leica Biosystems).

### Tumor efficacy studies

Mice-bearing surgically implanted tumors obtained from Black and White patients with sizes of 70–100 mm^3^ were randomized into groups as control, GemHCl, and 4NSG-SLN (*n* = 5/group). Baseline tumor volume was established, and dosing initiation began on day 1 with intravenous administration of 30 mg/kg Gem (twice weekly for 3 weeks) with an equivalent dose of GemHCl and 4NSG-SLN [42]. Once tumors became palpable, their volume was measured thrice per week, and the mice's weight was recorded twice weekly. Tumor volumes were measured using digital calipers and calculated using the following equation: V = (L*(W)^2^)/2, where V is the volume (mm^3^), W(width) is the smaller of two perpendicular tumor axes, and the L (length) is the larger of two vertical axes. Mean tumor volume growth curves were calculated for each treatment group [36].

#### Euthanization

After the tumor efficacy studies, mice were sacrificed by carbon dioxide (CO_2_), followed by decapitation. CO_2_ flow to the chamber was adjusted to 3 L per minute for 2 to 3 min and observed each mouse for lack of respiration and faded eye color. The CO_2_ flow was maintained for a minimum of 1 min after respiration ceased and followed by decapitation with scissors.

### Statistical analysis

The data were analyzed by GraphPad Software either by t-test or one-way ANOVA as indicated.

Pharmacokinetic parameters were estimated using the software PKSolver. The difference between GemHCl and 4NSG-SLN treatment groups was analyzed using Student's t-test and considered significant at *p* < 0.05. In vitro results are presented as ± SD, and in vivo results, as presented as mean ± SEM unless otherwise stated. All experiments were performed at least in triplicate and analyzed using GraphPad Prism software (GraphPad Software, Inc., San Diego, CA).

## Results

### Synthesis and characterization of 4NSG

4NSG was successfully synthesized based on the data obtained from NMR, micro elemental analysis, and mass spectrometer. The NMR spectra of 4NSG were observed using ^1^H and ^13^C NMR, and the results observed were consistent with reported data [24] (see Fig. 2a, SF1 (A-C)). The ^1^H NMR peaks were 10.88 ppm (-CO–NH), and 1.38 – 1.04 ppm (-CH_2_-)_15,_ representing the amide linkage and long-chain methylene group contributed by stearic acid, respectively. The formed amide bond in 4NSG revealed a characteristic ^13^C NMR peak at 174.55 ppm, confirming the presence of an amide bond between stearic acid and Gem. Based on micro elemental analysis results, (Calculated: C = 59.21; H = 8.65; *N* = 7.67; F = 6.94 compared to with Found: C = 59.27; H = 8.59; *N* = 7.51; F = 6.67), the purity of 4NSG was found to be 99.8%, and this result is similar to our HPLC purity analysis of 4NSG which was 99.8%. Mass spectrometer analysis of 4NSG was performed, and Supplementary Fig. 1C shows representative mass spectra obtained for 4NSG. The observed two fragments of 4NSG showed similar peak intensity, however; the m/z ratios of the fragments occurred at 530.59 and 1059.37. This corresponds closely to the molecular weight of 4NSG (530.59) and its respective dimer (1059.37).

### In vitro metabolic stability of 4NSG using human liver microsomes

In vitro metabolic stability of 4NSG using human liver microsomes was analyzed by HPLC with a detection wavelength of 268 nm. A graph of percent 4NSG stability against time revealed about 90% unchanged 4NSG present human liver microsomal solution. In comparison, the metabolic stability of 4NSG in PBS was found to be 99% at the end of the 4 h study (Fig. 2b.)

### Preparation and characterization of 4NSG-SLN

The interaction of the lipophilic end of 4NSG and labrasol, soy lecithin, and Tween 80 developed 4NSG-SLN. The TEM image of the formulated 4NSG-SLN appeared spherical with a narrow size distribution (Fig. 2c). Further, the hydrodynamic diameter (particle size) of 4NSG-SLN in PBS solution had a mean particle size of 82.3 ± 6.7 nm, and blank SLN was 35.1 ± 4.2 nm (Table 1, Supplementary Fig. 2). One important aspect of the formulation was determining the entrapment of 4NSG in the SLN delivery system. As expected, the entrapment efficiency was determined to be 98.7 ± 4.5%, while the zeta potential value of the dispersed 4NSG-SLN in PBS (10 mm, pH 7.4) was ± 11.8 mV (Table 1).

**Table 1:** Mean particle size, polydispersity index, and zeta potential of SLN and 4NSG-SLN formulation

Formulation	Particle size (nm)	Polydispersity Index	Zeta potential (mV)	Entrapment Efficiency (%)
SLN	35.1 ± 4.2	0.3	+ 0.2	-
4NSG-SLN	82.3 ± 6.7	0.5	+ 11.8	98.7 ± 4.5

Data showed are mean ± SD, *n* = 3 (- means no entrapment)

### In vitro stability of 4NSG-SLN based on hydrodynamic diameter and polydispersity index (PDI)

To evaluate the ability of 4NSG-SLN to remain dispersed in a solution devoid of aggregation or precipitation, in vitro stability of 4NSG-SLN in PBS or PBS + 25% FBS solution was conducted. The results showed an overall hydrodynamic diameter (particle size) ranging from 82 ± 2.0 nm to 89 ± 1.8 92 nm from 1^st^ day to 7^th^ day in PBS solution at 37 °C. While the overall hydrodynamic diameter for 4NSG-SLN in PBS + 25% FBS solution ranged from 82 ± 2.0 nm to 89 ± 1.8 nm for the 7-day study. No significant difference was observed between 4NSG-SLP hydrodynamic diameter size in PBS and PBS + 25% FBS solutions (Table 2).

**Table 2:** Stability of 4NSG-SLN based on hydrodynamic diameter and polydispersity index (PDI)

Day	Medium		Hydrodynamic diameter (nm)		PDI	
			PBS	PBS + 25% FBS	PBS	PBS + 25% FBS
1	PBS	PBS + 25% FBS	82 ± 2.0	84 ± 1.2	0.12 ± 0.08	0.17 ± 0.03
2	PBS	PBS + 25% FBS	83 ± 1.5	86 ± 3.6	0.26 ± 0.06	0.32 ± 0.07
3	PBS	PBS + 25% FBS	84 ± 4.2	88 ± 2.0	0.47 ± 0.05	0.45 ± 0.04
4	PBS	PBS + 25% FBS	85 ± 2.7	89 ± 1.6	0.38 ± 0.01	0.41 ± 0.01
5	PBS	PBS + 25% FBS	86 ± 3.1	90 ± 4.0	0.13 ± 0.04	0.37 ± 0.04
6	PBS	PBS + 25% FBS	87 ± 5.5	91 ± 2.1	0.26 ± 0.07	0.54 ± 0.02
7	PBS	PBS + 25% FBS	89 ± 1.8	92 ± 3.7	0.68 ± 0.06	0.71 ± 0.08

Data expressed as mean ± SD, *n* = 3 Hydrodynamic diameters (particle size)

### Evaluation of in vitro release of 4NSG -SLN

The cumulative in vitro drug release of 4NSG from 4NSG-SLN was determined for a 12 h period at 37 °C while maintaining sink condition. The rapid release of free 4NSG (Mol. weight of 547.68) from the dialysis bag (MCO of 3.5 kDa) was about 60% within the first 2 h and a further 20% increase in the next 2 h. No significant increase in free 4NSG release was observed after 4 h (Fig. 2d). For the freshly prepared 4NSG-SLN sample, a 30% release of 4NSG was observed within the first 2 h, followed by a gradual or slow release of 10% over the next 2 h. Further, a 10% release of 4NSG was observed from the 4 h to 12 h (Fig. 2d). For the lyophilized 4NSG-SLN sample, about 20% of 4NSG was released during the first 2 h period, followed by a slow release of an additional 15% within 6 h (Fig. 2d). There was no significant difference in the release of 4NSG between the freshly prepared 4NSG-SLN and lyophilized 4NSG-SLN samples.

### Cytotoxicity effect of GemHCl and 4NSG-SLN

The cytotoxic activity of 4NSG-SLN was evaluated in PPCL-192, PPCL-135, PPCL-46, and PPCL-68 cultures. After 48 h treatment, the 4NSG-SLN formulation was effective and significantly inhibited the growth of PPCL-192, PPCL-135, PPCL-46, and PPCL-68 cells compared to GemHCl treatments.

**Fig. 3:**
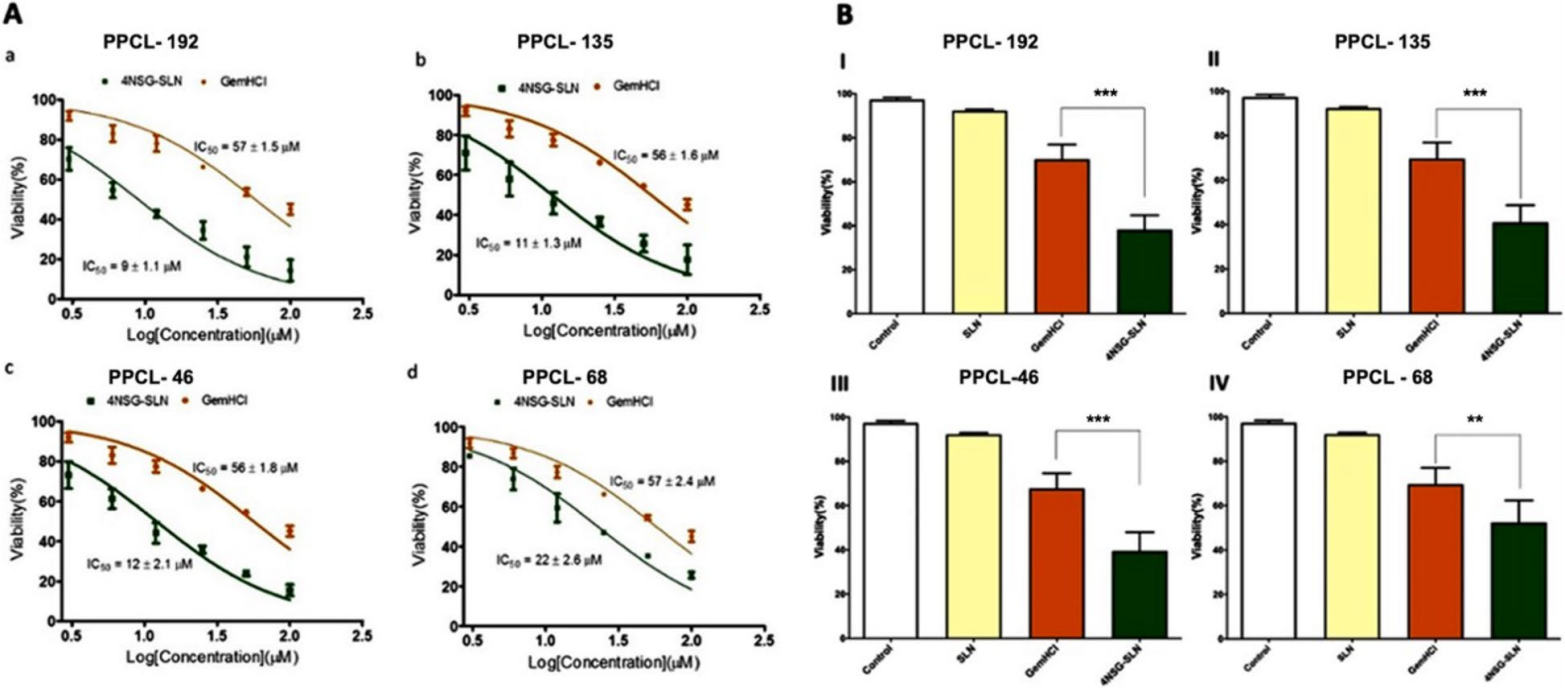
Cytotoxic activity of GemHCl and 4NSG-SLN against PPCL-192, PPCL-135, PPCL-46, and PPCL-68 cells at varying concentrations. IC_50_ values generated from dose–response non-linear curve fitting after treatment with GemHCl and 4NSG-SLN for (**A**) (a) PPCL-192 cells, (b) PPCL-135 cells, (c) PPCL-46 cells, and (d) PPCL-68 cells. **B** Viability of (I) PPCL-192, (II) PPCL-135, (III) PPCL-46, and PPCL-68 cells after treatment with GemHCl and 4NSG-SLN. Cell cultures treated with no drug set to 100% (controls) were used as a reference to evaluate the percent viability of vehicle (SLN), GemHCl, and 4NSG-SLN treated PPCL-192, PPCL-135, PPCL-46, and PPCL-68 cultures. Results represent at least three independent experiments and data expressed as mean ± standard deviation (SD), *n* = 3. Percent viability of (GemHCl vs. 4NSG-SLN, **p < 0.01, ***p < 0.001)

As shown in Fig. 3A and Fig. 3B, 4NSG-SLN demonstrated significant anticancer activity against PPCL-192, PPCL-135, PPCL-46, and PPCL-68 cultures with IC_50_ values significantly lower than the IC_50_ values of GemHCl-treated PPCL-192, PPCL-135, PPCL-46, and PPCL-68 cultures Table 3. Furthermore, an approximately 2.5 – sixfold decrease was observed in IC_50_ values in 4NSG-SLN treatments compared to GemHCl treated groups Table 3. The 4NSG-SLN treated PPCL-192 and PPCL-46 cells showed higher cytotoxic activity with IC_50_ values of 9 ± 1.1 µM and 12 ± 2.1 µM respectively, compared to their GemHCl treatments, 57 ± 1.5 µM for PPCL-192 and 56 ± 1.8 µM for PPCL-46. Based on their cytotoxicity activity PPCL-192 and PPCL-46 cells were chosen for further studies in this study.

**Fig. 4:**
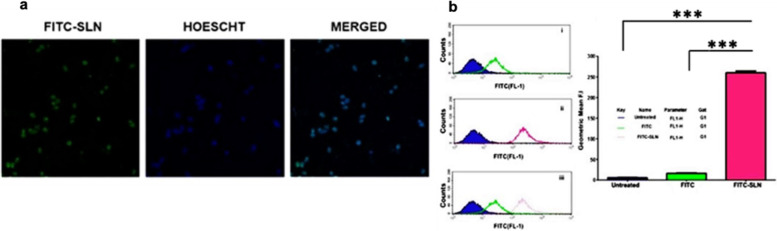
Cellular Uptake: (**a**) Confocal microscopy images showing cellular uptake of FITC-SLN by PPCL-192 after incubation for 3 h. The nucleus was stained using the DNA-binding dye Hoechst 33,342 (shown in blue) after cell fixation and the presence of FITC-SLN in cells labeled in green. The merged image exhibits the co-localization of FITC-SLN and Hoechst 33,342 in the nucleus of PPCL-192 cells (Scale bar = 10 µm). **b** Flow cytometry analysis of PPCL-192 cellular uptake of FITC and FITC-SLN compared to untreated (control) cells after 4 h of incubation. The geometric mean of PPCL-192 cellular uptake of FITC and FITC-SLN. (***p < 0.001 for comparing the geometric mean of untreated, FITC-treated, and FITC-SLN-treated cells) Untreated = negative control; FITC = positive control and FITC-SLN = cells exposed to FITC-SLN, *n = *3

**Table 3:** IC_50_ values of GemHCl and 4NSG-SLN treated PPCL-46, PPCL-68, PPCL-135, and PPCL-192 cultures

	**IC**_**50**_** μM** **(PPCL-46)**	**IC**_**50**_** μM** **(PPCL-68)**	**IC**_**50**_** μM** **(PPCL-135)**	**IC**_**50**_** μM** **(PPCL-192)**
GemHCl	56 ± 1.8	57 ± 2.4	56 ± 1.5	57 ± 1.5
4NSG-SLN	12 ± 2.1	22 ± 2.6	11 ± 1.3	9 ± 1.1

Data represent ± SEM, *n* = 3

### Cellular uptake of FITC-labeled SLN

#### Confocal imaging

Cellular uptake of the 4NSG-SLN formulation was determined by treating PPCL-192 and PPCL-46 for 3 h at 37 °C with FITC-conjugated SLN. Confocal images of PPCL-192 Fig. 4a showed significant uptake of FITC-SLN counterstained with Hoechst nuclei dye. The merged images indicated that most internalized nanoparticles were localized in the cells’ nuclei.

### Flow cytometry

A flow cytometry study was conducted to confirm further the internalization of the FITC-conjugated SLN against PPCL-192 and PPCL-46 cells. After 24 h exposure of both cells to FITC-SLN, there was a significant cellular uptake with about a tenfold increase in geometric mean fluorescence intensity observed in FITC-SLN exposed PPCL-192 and PPCL-46 cells compared to FITC as shown in Fig. 4b.

### Clonogenic survival assay

**Fig. 5:**
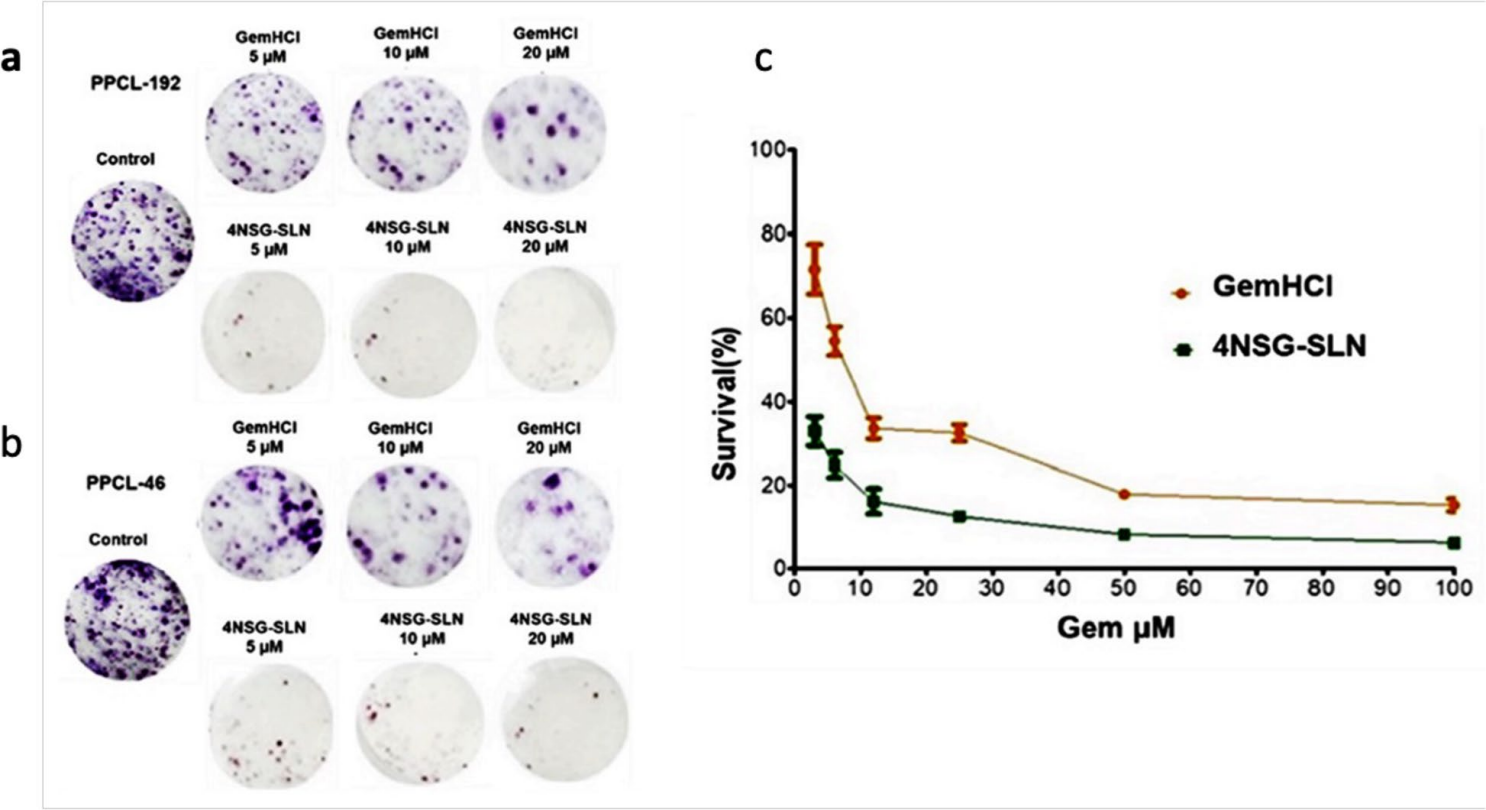
Colony formation studies of GemHCl and 4NSG-SLN against PCa cells (**a**) Colony formation assay post-treatment images of GemHCl and 4NSG-SLN treated PPCL-192, (**b**) Colony formation assay post-treatment images of GemHCl and 4NSG-SLN treated PPCL-46, (**c**) Survival curve of GemHCl and 4NSG-SLN treated PCa cells. Data represent mean ± SD, *n* = 3 with 20X magnification

The colony formation assay assessed the proliferative property of PPCL-192 and PPCL-46 after exposure to varying treatment concentrations of GemHCl and 4NSG-SLN. The difference in percent survival of GemHCl and 4NSG-SLN treated PPCL-192 and PPCL-46 cells showed a colony formation reduction in 4NSG-SLN treated cells at varying concentrations. This confirms 4NSG-SLN’s disrupting proliferative property against cancer cells compared to GemHCl (Fig. 5a and 5b). The survival curve follows a similar trend, as shown in (Fig. 5c). The results obtained from the colony assay indicate the effectiveness of 4NSG-SLN and its ability to significantly reduce cell survival and proliferation than GemHCl.

### Cell cycle analysis

**Fig. 6:**
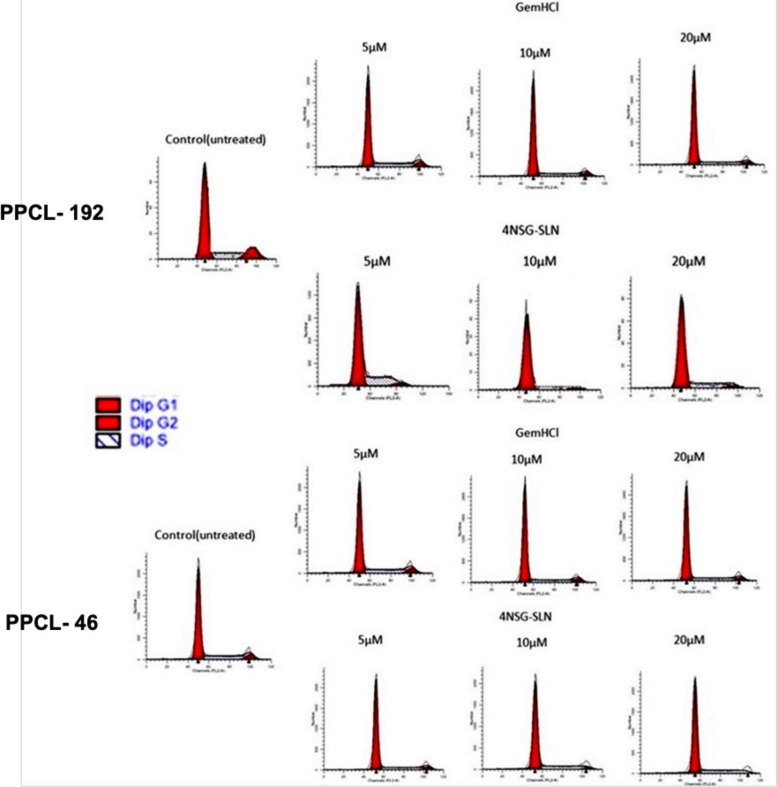
Cell Cycle: The effect of 4NSG-SLN and GemHCl on cell cycle distribution in PPCL-192 and PPCL-46 cells. Both cells were treated with 4NSG-SLN and GemHCl at (5 μM, 10 μM, and 20 μΜ) for 24 h and stained with PI to analyze the cell cycle distribution of each cell type by flow cytometry. The percent cell cycle distribution is relative to the total phases (G1, S, and G2). 4NSG-SLN induces G1-phase cell cycle arrest at low concentrations (5 μM) and consequently, 4NSG-SLN treatment at higher concentrations (20 μM) induced cell death in PPCL-192 and PPCL-46 cells compared to GemHCl treatment groups

Cell cycle analysis was performed to determine the ability of 4NSG-SLN to stop cell cycle progression by arresting cell division at certain checkpoints responsible for promoting apoptosis. PPCL-192 and PPCL-46 cells were treated with 4NSG-SLN formulation with GemHCl equivalence at concentrations (5 μM, 10 μM, and 20 μM) for 24 h. Figure 6 revealed a typical DNA pattern representing the cell cycle's G1, S, and G2 phases were observed. At 5 μM concentration, 4NSG-SLN treated cells showed a higher G1 population (80.57%) and (78.25%) in PPCL-192 and PPCL-46 cells, respectively, compared with their corresponding GemHCl treatments (71.08%) for PPCL-192 cells and (72.75%) for PPCL-46 cells. At 5 μM concentration, 4NSG-SLN treated cells revealed a higher G1 population (80.57%) and (78.25%) in PPCL-192 and PPCL-46 cells, respectively, compared with their corresponding GemHCl treatments (71.08%) for PPCL-192 cells and (72.75%) for PPCL-46 cells. The 4NSG-SLN treated groups revealed a concomitant reduction in the proportion of cells in the S and G2 phases in PPCL-192 and PPCL46 cells (Table 4). The 4NSG-SLN showed a decrease in cell population entering the G2-phase at 20 µM concentration in PPCL-192 and PPCL-46 treated cells at (3.18%) and (2.45%) respectively, compared to GemHCl treatments which showed a higher cell population of (8.23%) and (9.54) for PPCL-192 and PPCL-46 respectively. The cell cycle data demonstrated that 4NSG-SLN induced G1-phase cell cycle arrest at low concentrations (5 μM) and higher concentrations (10 μM and 20 μM) induced cell death in PPCL-192 and PPCL-46 cells.

**Table 4:** Percent cell cycle distribution of GemHCl and 4NSG-SLN in PPCL-92 and PPCL-46 cells

Cell line		GemHCl			4NSG-SLN		
		%G1	%S	%G2	%G1	%S	%G2
PPCL-192	Control (untreated)	66.47	11.07	18.46	58.75	11.07	18.46
	5 μΜ	71.08	17.87	11.05	80.57	12.22	7.21
	10 μM	74.75	15.66	9.59	83.25	11.44	5.31
	20 μM	79.91	11.86	8.23	90.45	6.37	3.18
PPCL-46	Control (untreated)	68.75	10.90	20.35	68.75	10.90	20.35
	5 μΜ	72.75	14.69	12.56	78.25	11.61	10.14
	10 μM	74.08	15.53	10.39	82.12	10.49	7.39
	20 μM	76.35	14.11	9.54	87.53	10.02	2.45

Data represent ± SEM, *n* = 3

### 4NSG-SLN cell migration analysis

**Fig. 7:**
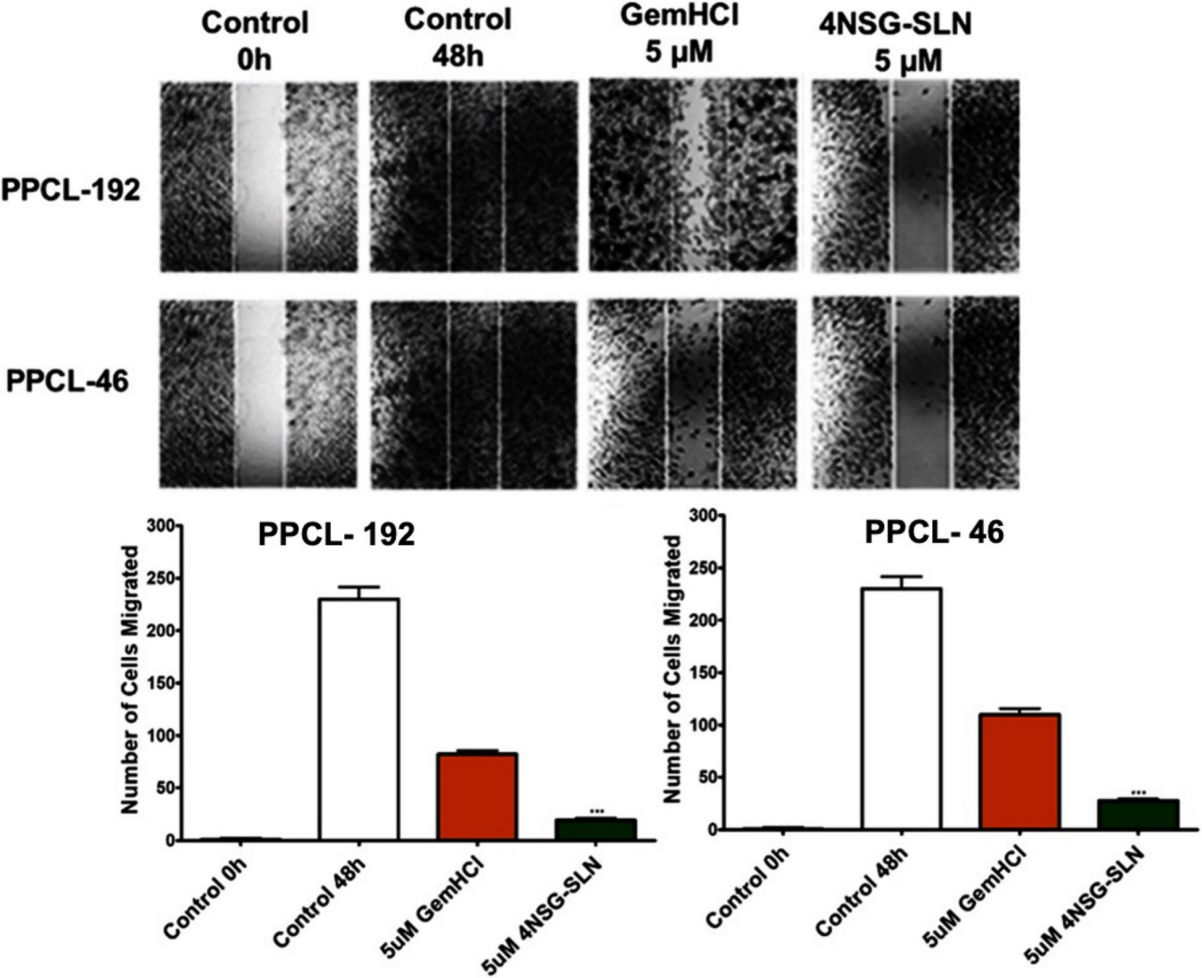
Migration Studies. The wound healing method determined the effect of GemHCl and 4NSG-SLN on PPCL-192 and PPCL-46 cell migration (**a**) images of GemHCl and 4NSG-SLN treated PPCL-192 and PPCL-46 cells at 5 µM concentration towards the scratch wound. NIH ImageJ software quantified and analyzed the average number of (**b**) PPCL-192 and (**c**) PPCL-46 migrated cells. Wound closures were captured at 10X magnification using the Nikon Eclipse Ti inverted fluorescent microscope. The plots are of the means ± SD, *n* = 3. Significance (GemHCl and 4NSG-SLN ***p < 0.0001) was determined by t-test

Cell migration assay examined cell mobility towards a uniform "wound" made in confluent monolayer culture of PPCL-192 and PPCL-46 cells. The number of cells that migrated towards the wound was evaluated at 5 µM after 48 h. The control (untreated) PPCL-192 and PPCL-46 cells, after 48 h, migrated towards the wound, covering almost the entire surface area of the Ibidi cell culture inserts (Fig. 7). 4NSG-SLN treated PPCL-192 and PPCL-46 cells at 5 μΜ concentration significantly reduced cell mobility towards the wound area with (18 ± 2.5) and (28 ± 3.5) number of cells migrated respectively compared to GemHCl treatments which showed (84 ± 3.1) for PPCL-192 and (120 ± 3.8) for PPCL-46 as indicated in (Fig. 7 and Table 5).

**Table 5:** Quantification of GemHCl and 4NSG-SLN treated cells that migrated toward the scratched area

Number of cells migrated				
Concentration (µM)	**PPCL-192**		**PPCL-46**	
	GemHCl	4NSG-SLN	GemHCl	4NSG-SLN
0 (control)	261 ± 6.4	261 ± 6.4	261 ± 2.8	261 ± 2.8
5	84 ± 3.1	18 ± 2.5	120 ± 3.8	28 ± 3.5

Data represent ± SEM, *n* = 3

### Pharmacokinetic study of 4NSGSLN

The pharmacokinetic parameters of 4NSG-SLN were determined by administering a 20 mg/kg bolus intravenous injection in mice. An equivalent dose of GemHCl was also administered to mice using the same route to evaluate and compare their pharmacokinetic parameters. As indicated in Table 6, the half-life of 4NSG-SLN (1.93 ± 0.06 h) was threefold higher compared with GemHCl (0.70 ± 0.01 h) with a *p*-value of 0.0001. The AUC also showed a similar trend where 4NSG-SLN (86.2 ± 5.43 µg/mL*h) showed a threefold increase in bioavailability compared to GemHCl (28.2 ± 4.3 µg/mL*h) with a *p*-value of 0.0001. Since half-life is inversely proportional to plasma clearance, it was observed that an increase in the half-life resulted in a decrease in clearance for 4NSG-SLN (7.1 ± 1.1 mL/h) compared with an increase in clearance for GemHCl (21.3 ± 3.2 mL/h) with a *p*-value of 0.0001.

**Table 6:** Pharmacokinetic profiles after GemHCl and 4NSGSLN were administered intravenously to mice

Parameter	Unit	Comparison		*p*-value
		**GemHCl**	**4NSG-SLN**	
k_10_	1/h	1.01 ± 0.03	0.36 ± 0.04	0.0001
t_1/2_	H	0.70 ± 0.01	1.93 ± 0.06	0.0001
V_d_	mL	21.7 ± 1.9	19.3 ± 0.2	0.3619
Cl	mL/h	21.3 ± 3.2	7.1 ± 1.1	0.0001
AUC_(0-t)_	µg/(mL*h)	28.2 ± 4.3	86.2 ± 5.4	0.0001

K_10_, elimination rate constant; t_1/2_, half-life; AUC_(0-t),_ area under the plasma concentration–time curve; *Cl* Clearance, *V*_*d*_ Volume of distribution; (Data analyzed using t-test)

### Tumor efficacy studies of 4NSG-SLN

**Fig. 8:**
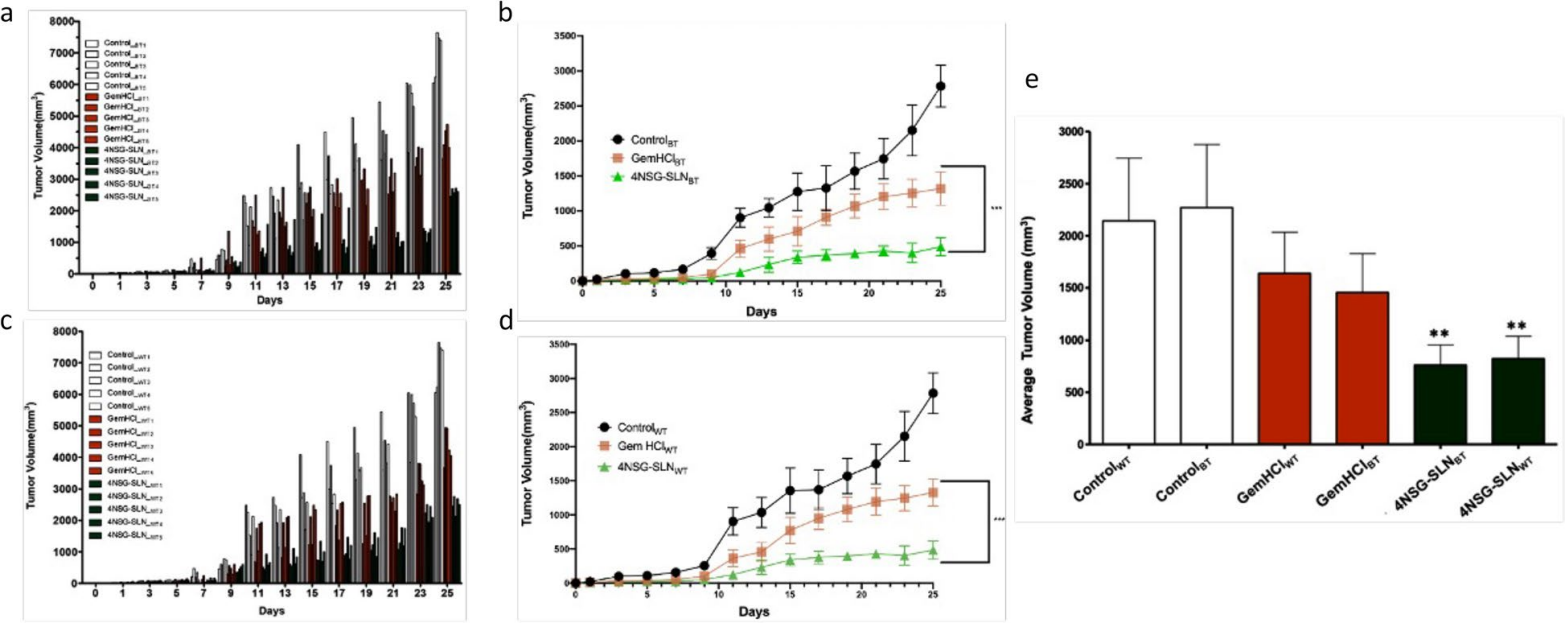
Tumor volume growth for individual PDX mice in each treatment group. **a** Normalized tumor volume of GemHCl and 4NSG-SLN from Black (BT) PCa patients (*n* = 5), (**b**) Tumor growth curves of GemHCl and 4NSG-SLN treated mice bearing PCa PDX tumor from Black patients, (**c**) Normalized tumor volume of GemHCl and 4NSG-SLN from White (WT) PCa patients (*n* = 5), (**b**) Tumor growth curves of GemHCl and 4NSG-SLN treated mice bearing PCa PDX tumor from White patients, (**d**) mean terminal tumor growth volumes of control, GemHCl, and 4NSG-SLN treatment groups of PDX mice bearing tumors from Black and White PCa patients. Data represented as means ± SD, *n* = 5. The significance difference (GemHCl vs 4NSG-SLN, **p < 0.001) was determined by one-way ANOVA

Tumor heterogeneity is evident in PCa patients. To mimic this in our PDX study, each mouse-bearing tumor from Black and White PCa patients was treated individually to assess the antitumor activity of 4NSG-SLN compared to GemHCl treatments for 25 days. The normalized tumor volume of range between (300 mm^3^ to 800 mm^3^) was observed in 4NSG-SLN treated mice bearing tumors from Black patients on day 11, indicating a significant tumor inhibition compared to GemHCl treatments which were within the range of (1100 mm^3^ to 1700 mm^3^). A similar trend was observed on days (13 through 25) for 4NSG-SLN treatments, which showed an overall increase in antitumor efficacy in individual mice bearing PCa tumor from Black patients, as indicated in Fig. 8a. The 4NSG-SLN formulation inhibited tumor growth in PDX mice bearing tumors from White patients on day 11 of this study. The normalized tumor volume ranging from (400 mm^3^) in 4NSG-SLN mice bearing White’s tumor compared with that of GemHCl treatments ranging from (1200 mm^3^ to 2200 mm^3^) confirms a similar trend observed in days (13 through 25) as shown in Fig. 8c. The antitumor activity of 4NSG-SLN was further assessed by comparing average terminal tumor inhibition in both Black and White PDX mice Fig. 8e, Table 7 with GemHCl treatments and control. The 4NSG-SLN treatments continuously showed decreased tumor growth for individual PDX mice bearing tumors obtained from Blacks and Whites. The mean tumor volume for the control group from both PDXs from Black and White PCa tumors was extremely large, but 4NSG-SLN exhibited a significant decrease in tumor growth, as observed in (Fig. 8b and Fig. 8d).

**Table 7:** Comparison of average terminal tumor volumes of GemHCl and 4NSG-SLN treated mice bearing PCa tumors obtained from Black and White pancreatic cancer patients

Day	Mean terminal tumor volume (mm^3^)					
	GemHCl_BT_	GemHCl_WT_	*p*-value^ǂ^	4NSG-SLN_BT_	4NSG-SLN_WT_	*p*-value^¤^
0	0	0	Ns	0	0	0
1	12.5 ± 6	13.9 ± 2	ns	12.6 ± 2	16 ± 3	0.004
3	25.0 ± 12	27.8 ± 4	ns	25.3 ± 3	32 ± 5	0.004
5	35.7 ± 18	39.7 ± 5	ns	36.2 ± 4	46.1 ± 9	0.004
7	73.0 ± 21	43.5 ± 16	ns	51.2 ± 9	50.7 ± 17	ns
9	229. ± 181	177.8 ± 60	ns	129 ± 35	182 ± 44	0.03
11	662. ± 198	583.2 ± 227	ns	341.4 ± 166	247.8 ± 76	ns
13	767. ± 194	646.9 ± 240	ns	375.6 ± 182	300 ± 90	ns
15	911. ± 152	752.6 ± 241	ns	418 ± 196	359.4 ± 108	ns
17	990. ± 151	850.8 ± 205	ns	449 ± 226	421 ± 112	ns
19	1129 ± 172	868.2 ± 294	ns	432 ± 105	494.8 ± 121	ns
21	1204 ± 184	1053 ± 86	0.05	429.3 ± 68	564.5 ± 128	ns
23	1456 ± 154	1344.7 ± 171	ns	521.7 ± 71	896.3 ± 93	0.0003
25	1681 ± 172	1748.2 ± 223	ns	1044.5 ± 40	1002.7 ± 96	ns

Data expressed in mean ± SD, *n* = 5

### Immunohistochemistry (IHC) Studies of 4NSG-SLN

**Fig. 9:**
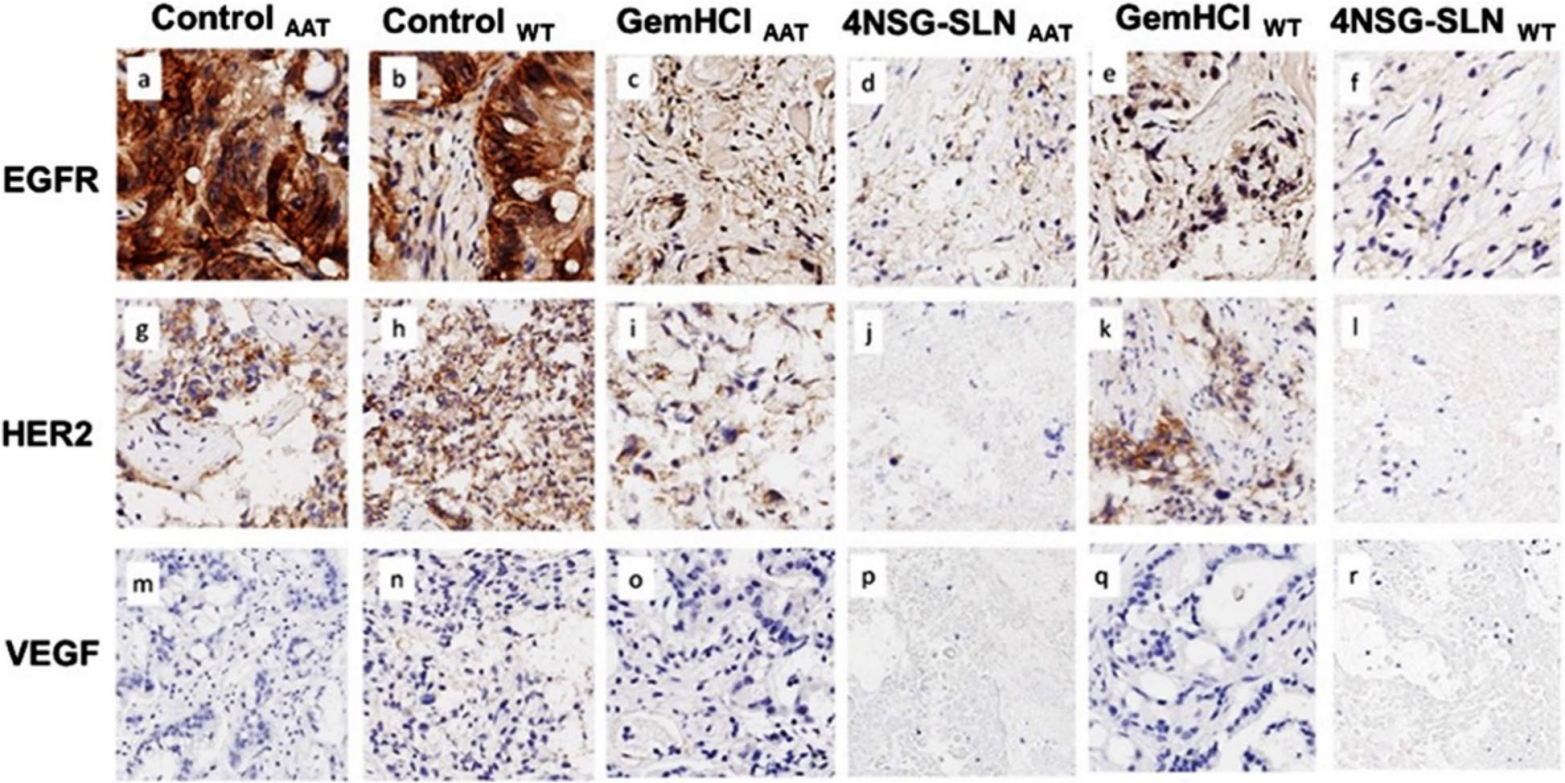
Expression of EGFR, HER2, and VEGFR receptors in GemHCl and 4NSG treated mice bearing PDX PCa tumor tissues from Black and White PCa patients**.** Representative images of 4NSG-SLN_BT_ and 4NSG-SLNWT staining showed moderate EGFR expression (d and f) but negative staining for HER2 and VEGFR (j, l, p, and r). GemHCl_BT_ and GemHCl_WT_ revealed moderate to high EGFR, HER2, and VEGFR expressions (c, e, I, k, o, q) with 40X magnification. Representative images (a,g, and m) represented control _AAT_ receptor expressions for EGFR, HER2, and VEGF, respectively, and images (b,h.n) represented control _WT_ receptor expressions for EGFR, HER2, and VEGF, respectively

The expressions of EGFR, HER2, and VEGFR were evaluated in mice pancreatic tumor tissues using EGFR, HER2, and VEGFR staining. Positive membrane staining was observed in EGFR (Control_BT_ = 25 ± 2.3, Control_WT_ = 26.3 ± 4.4), HER2 (Control_BT_ = 18.4 ± 0.6 Control_WT_ = 27.3 ± 2.3) and VEGFR ( Control_BT_ = 33.6 ± 4.3, Control_WT_ = 35.2 ± 0.1). The impact of the 4NSG-SLN formulation was evaluated on EGFR, HER2, and VEGFR expressions in the mice's pancreatic tumor tissue. Figure 9 shows immunohistochemical staining of PDX tumor tissues with positive staining for EGFR (4NSG-SLN_BT_ = 14.2 ± 5.5, 4NSG-SLN_WT_ = 5 ± 2.2), HER2 4NSG-SLN_BT_ = 2.3 ± 1.2 4NSG-SLN_WT_ = 3.6 ± 1.9) and VEGFR (4NSG-SLN_BT_ = 6.6 ± 1.8, 4NSG-SLN_WT_ = 7.7 ± 1.4). There was a significant reduction in EGFR, HER2 and VEGFR expressions in both 4NSG-SLN treated mice bearing Black and White tumor expressions as observed in Fig. 9. The difference in immunoreactivity intensity of GemHCl and 4NSG-SLN treated tumor tissue obtained from Black’s and White’s tumors were further observed, and stained sections were analyzed as shown in Table 8.

**Table 8:** Differential receptor expression in PDX mice bearing Black and White pancreatic cancer tumors after treatment with GemHCl and 4NSG-SLN with their respective staining scores analysis

	**Control**_**BT**_	**Control**_**WT**_	**GemHCl**_**BT**_	**4NSG-SLN **_**BT**_	***p*-value**^Υ^	**GemHCl**_**WT**_	**4NSG-SLN**_**WT**_	***p*-value**^‡^
**EGFR**								
% Positive	25 ± 2.3	26.3 ± 4.4	25 ± 4.5	14.2 ± 5.5	0.043	20 ± 10	5 ± 2.2	0.562
% Weak Positive Cells	1.3 ± 0.1	1.9 ± 1.3	1.4 ± 0.1	7.3 ± 2.9		2.7 ± 0.7	2.7 ± 1 ± .4	
% Moderate Positive Cells	0.3 ± 0.1	0.5 ± 0.4	0.6 ± 0.2	3.1 ± 2.1		3.1 ± 2.1	0.9 ± 0.7	
% Strong Positive Cells	0.4 ± 0.1	0.4 ± 0.1	0.5 ± 0.1	3.8 ± 0.5		2.3 ± 1.5	0.8 ± 0.1	
%Negative Cells	73 ± 3.1	70.9 ± 0.2	72.5 ± 1.5	71.6 ± 2.4		71.9 ± 1.6	90.6 ± 0.9	
H Score	10.4	6.2	3.0	31.0		22.1	9.4	
**HER2**								
% Positive	18.4 ± 0.6	27.3 ± 2.3	17.2 ± 1.0	2.3 ± 1.2	0.020	19.9 ± 1.5	3.6 ± 1.9	0.008
% Weak Positive Cells	26.8 ± 0.8	11.2 ± 1.3	9.3 ± 1.4	11.8 ± 0.9		8.9 ± 0.8	2.7 ± 1.4	
% Moderate Positive Cells	12 ± 1.0	4.1 ± 1.2	7.2 ± 4.3	0.07 ± 0.002		10 .2 ± 1.1	0.03 ± 0.01	
% Strong Positive Cells	5.2 ± 0.6	6.3 ± 2.6	3.7 ± 0.8	1.6 ± 0.7		3.6 ± 1.2	1.5 ± 0.74	
%Negative Cells	37.6 ± 1.9	51.1 ± 0.8	62.6 ± 3.4	84.2 ± 6.1		57.4 ± 7.3	92.2 ± 6.1	
H Score	48.5	50.1	31.8	20.2		30.9	11.6	
**VEGF**								
% Positive	33.6 ± 4.3	35.2 ± 0.1	27 ± 0.4	6.6 ± 1.8	0.010	30 ± 1.4	7.7 ± 1.4	0.006
% Weak Positive Cells	1.8 ± 0.2	0.3 ± 0.1	1.2 ± 0.3	2.7 ± 1.4		0.7 ± 0.3	0.3 ± 0.003	
% Moderate Positive Cells	0.5 ± 0.1	0.2 ± 0.1	0.6 ± 0.2	0.4 ± 0.01		0.6 ± 0.1	0.4 ± 0.2	
% Strong Positive Cells	0.6 ± 0.2	0.4 ± 0.1	0.5 ± 0.1	0.85 ± 0.1		0.7 ± 0.3	0.5 ± 0.3	
% Strong Positive Cells	63.5 ± 1.7	63.9 ± 1.2	70.7 ± 7.8	89.5 ± 2.8		68 ± 3.8	91.1 ± 8.1	
H Score	16.1	36.6	4.7	1.4		10.0	1.8	

Data expressed in mean ± SD, *n* = 5 Data expressed in mean ± SD, *n* = 5

## Discussion

Numerous investigations of commercially available cells have demonstrated Gem's effectiveness in suppressing tumor growth and overcoming resistance significantly. Although monogenous cell lines lack predictive value, we cannot ignore their relevance in research as they still play an important role in the preliminary screening of anticancer agents. PPCLs obtained from patient-derived xenografts (PDX) preserve in part the intratumoral heterogeneity that exists in pancreatic cancer is the new approach adopted for screening chemotherapeutic agents. The expansion of PPCLs from PDXs has been successful in 100% of attempts. Therefore, there is a need to utilize primary cells such as PPCL-135 and PPCL-192, PPCL-46 PPCL-68 from patient-derived xenografts, demonstrating highly reproducible, predictive, and translational values [21, 43, 44],. Results from SEER show a higher incidence of PCa in the Black and White PCa population. Therefore, performing a high throughput screening of 4NSGSLN against primary cells and PDXs obtained from these two populations is vital. This current study highlights the importance of evaluating the anticancer activity of 4NSG-SLN on patient-derived PCa cells in vitro using (PPCL -192, PPCL-135) and (PPCL-46, PPCL-68) obtained from Black and White patients, respectively, and further assesses the antitumor efficacy in PCa PDX mice bearing tumors from Blacks and Whites. Subcutaneous PDX model was used to assess the antitumor efficacy of 4NSG-SLN compared with GemHCl. In our immediate future studies, we plan to compare the anticancer activities of GemHCl, 4NSG, 4NSG-SLN and surface-modified targeted 4NSG-SLN in orthotopic PDX models. It is important to note that others have demonstrated the effectiveness of some Gem analogs in several commercial cell lines [24, 45] but there’s little information available comparing the efficacy of Gem analogs in patient-derived PCa cells from Black and White patients. This research bridges the gap and provides scientific studies supporting the benefits of using patient-derived PCa cells to evaluate the efficacy of Gem analogs in Blacks and Whites.

Before formulation and in vitro studies, the structure and purity of 4NSG were confirmed using NMR, micro-elemental analysis, HPLC, and mass spectrometry. The purity of the 4NSG obtained suggests that the synthesized compound used in this study was pure and was significantly devoid of any unwanted material [22].

Human liver microsome was used to determine the in vitro metabolic stability of 4NSG. We found that 4NSG was very stable and would likely remain intact or unchanged in the system circulation compared to GemHCl as reported in literature [46, 47].

Pharmaceutical nano-drug delivery systems have been employed to target anticancer drugs to tumors of interest and reduce unintended distribution and adverse effects on healthy cells. Nano-drug delivery systems enhance drug properties, such as increased entrapment and prolonged systemic circulation.

This protects anticancer drugs from first-pass metabolism and enzymatic degradation. SLNs possess biocompatible and biodegradable properties, which shield encapsulated drugs from harsh conditions [48]. Recent studies in nano-drug delivery approaches show a controlled drug release and an improved cellular uptake of SLN formulation in several organs and tumors compared to Gem [49]. Owing to the reasons above, an unconventional SLN capable of delivering a high payload of Gem was developed to improve its therapeutic efficacy against PCa cells. The cold homogenization method was used in formulating 4NSG-SLN to overcome temperature-induced drug degradation, drug distribution into the aqueous phase, and crystallization modification [29]. The choice of surfactants tremendously impacts SLN particle size. The surfactant compositions of lecithin, labrasol, and Tween 80 were kept below 2% to produce SLN with smaller particle size and carter for toxicity issues associated with elevated surfactant concentrations [50]. The mean particle size reduction in 4NSG-SLN (Table 1) was confirmed using a TEM image of the 4NSG-SLN formulation, which showed spherical nanoparticles, as indicated in Fig. 2c. The stability of drug-loaded solid-lipid nanoparticles in a biological medium is an important aspect to monitor in drug formulation. This study showed a successful formulation and characterization of 4NSG-SLN but in future studies we will perform a scale-up production of 4NSG-SLN utilizing high-pressure homogenization coupled with circulating production mode. The 4NSG-SLN stability studies via hydrodynamic particle size measurement indicated a slight size increase over 7 days (Table 2). This may suggest 4NSG-SLN’s ability to retain its physicochemical stability in circulation behavior in vivo. The dialysis bag method was used to determine the cumulative in vitro release of 4NSG from Freshly prepared and Lyophilized 4NSG-SLN formulations. These formulations were designed to encapsulate and retain 4NSG within the SLNs. The fast release of a significant percentage of free 4NSG from the dialysis bag could be attributed to the rapid diffusion observed within the first 2 h of the study. The gradual or slow release of 4NSG from the lyophilized and Freshly prepared 4NSG-SLNs implies that most of the 4NSG remained entrapped in the SLNs.

4NSG-SLN treatments against PPCL-192, PPCL-135, PPCL-46, and PPCL-68 cultures showed a higher antiproliferative activity compared to GemHCl. This might be attributed to the exceptional characteristics of 4NSG-SLN due to the modification of the polar nature of GemHCl to moderately lipophilic through the conjugation of Gem to stearic acid. Additionally, Gem has been reported to rely heavily on nucleoside transporters (hENT1) to deliver and accumulate into PCa cells. Under-expression of these transporters has been demonstrated to confer resistance to PCa [51]. Our results show that incorporating 4NSG into SLN indicates its ability to decrease PCa cell proliferation and suggests a potential anticancer agent for pancreatic cancer. Based on our research findings, we hypothesize that 4NSG-SLN can enter tumor cells by clathrin-mediated endocytosis.

During this process, lysosomal enzymes catalyze the degradation of the SLN, and the degradation facilitates the release of 4NSG from the nanoparticles. In the lysosome, enzymes such as cathepsin B catalyze the hydrolysis of 4NSG into Gem. The released Gem is exported from the lysosome into an appropriate intracellular compartment in the cytoplasm by nucleoside transporters such as lysosome-specific hENT3. Gem present in the cytoplasm undergoes phosphorylation and is converted into its active metabolites, dFdCDP and dFdCTP. 4NSG can diffuse and be transported into tumor cells via passive diffusion. However, due to the highly lipophilic nature of 4NSG, the release of Gem from 4NSG after hydrolysis might occur outside the suitable intracellular compartment for efficient phosphorylation. This might result in the likelihood of Gem undergoing deamination by cytidine deaminase, considering that nucleotides usually do not enter cells in the form of long-chain fatty acid conjugates [52, 53],.

The cellular uptake of 4NSG-SLN was evaluated using flow cytometry and confocal imaging. Flow cytometry and confocal studies indicated a tenfold increase in geometric fluorescent intensity in FITC-SLN compared to FITC-exposed cells. This suggests that the SLN could entrap a higher payload of 4NSG, which confirms the increase in the intensity of FITC-SLN compared to FITC alone.

The clonogenic assay measures the ability of the cells to proliferate and metastasize in forming colonies of at least 50 or more cells with a critical metric of cell viability [54]. The data suggest that 4NSG-SLN was more effective in rendering the cells incapable of proliferation than GemHCl Fig. 5 (a to c). The role of 4NSG-SLN in cell mobility was explored by conducting a cell migration experiment using PPCL-192 and PPCL-46 cells. Our findings revealed that 4NSG-SLN significantly reduced cell migration compared to GemHCl in both cells.

The regulation of cell cycle progression in cancer cells is considered a potentially effective strategy for controlling tumor growth [55]. Most cancer-related diseases are under frequent mutation, making the molecular analysis of cell cycle regulators vital [56]. With reference to the literature, GEM is a cell cycle-specific anticancer agent. It can arrest the cell cycle at the G1 phase, inhibiting DNA synthesis at the S phase. Our research findings for cycle cell indicated that 4NSG-SLN treated PPCL-192 and PPCL-46 cells at 5μΜ concentration resulted in a significant G1-phase arrest of cell cycle progression. The G1-phase cell cycle arrest observed at 5 μΜ concentration suggests a cell-cycle specific nature observed in Gem treatments. The cell cycle arrest at G1-phase revealed in our study could account for an increase in number of cells at the G1-phase which prevented the progression of cells into the S-phase to undergone DNA synthesis [57]. Previously, we demonstrated 4NSG’s ability to induce early apoptosis in pancreatic cancer cell at higher treatment concentrations [58].

Therefore, 4NSG-SLN may have triggered apoptosis in the PPCL-192 and PPCL-46 cells at 20μΜ concentration which confirms the least percent cell population accumulated studies' G2/M phases (Fig. 6, Table 4). Our cell cycle studies' results further support our assertion that 4NSG-SLN exhibited a more potent cytotoxic effect than GemHCl. The cell migration process is essential in driving tumor metastasis. In this study, 4NSG-SLN suppressed cellular mobility in PPCL-192 and PPCL-46 cells. The 4NSG-SLN significantly reduces cellular migration compared to GemHCl. The 4NSG-SLN formulation at 5 μM concentration, very few cells were present in the wound region, which indicated the ability of 4NSG-SLN to suppress cellular mobility compared to GemHCl. Comparing the pharmacokinetic profiles of 4NSG-SLN compared to that of GemHCl, it was observed that the half-life and AUC parameters significantly increased the residence time and the percentage of 4NSG-SLN that remained intact in systemic circulation compared to GemHCl.

EGFR is overexpressed in 30–89% of pancreatic cancer, and its role in predicting prognosis remains controversial [59, 60]. HER2 overexpression is seen in 4–50% of pancreatic cancer cases, and like EGFR, its role in predicting prognosis also remains unclear [61]. In some patients with PCa, HER2 expression predicts a worse prognosis, whiles for others; no association was found [11, 62]. VEGFR overexpression, on the other hand, is a good predictor of advanced stage and recurrence after resection in PCa [12].

In this study, we investigated the expressions of EGFR, HER2, and VEGFR in GemHCl and 4NSG-SLN-treated PDX mouse models bearing pancreatic tumors. In this particular PCa PDX mouse model obtained from Black and White patients, we observed lower expressions of EGFR, HER2, and VEGFR after IHC staining tumor of 4NSG-SLN_BT_ and 4NSG-SLN_WT_ treated groups suggesting that 4NSG-SLN may not effectively suppress the expression of these receptors, however; they may be targeted for drug delivery [63, 64],. A significant decrease in EGFR, HER2, and VEGFR receptor expressions of 4NSG-SLN was observed compared to GemHCl treatments in Black’s and White tumor tissues. Clinical data indicate that targeting EGFR, HER2, and VEGFR receptors may circumvent acquired tumor resistance and improve pancreatic cancer therapeutic outcomes. Based on this, we could suggest that 4NSG-SLN may have resulted in a significant decrease in tumor growth and is most likely due to 4NSG's ability to target EGFR, HER2, and VEGFR receptors.

Antitumor activity of 4NSG-SLN conducted over 25 days exhibited significant tumor growth inhibition compared with GemHCl in both Black’s and White’s tumors. The SLN may play an excellent role in encapsulating a higher payload of 4NSG. This led to high accumulation in the tumor site to cause a substantial tumor inhibition observed.in mice bearing Black’s and White’s tumors throughout the study period. Some critical factors could be used to explain the extraordinary tumor efficacy of 4NSG-SLN: i) absence of free NH_2_-group on 4NSG, which rendered the cysteine deaminase enzyme ineffective in metabolizing Gem. This event most likely allowed for prolonging circulation, increased bioavailability, and improved therapeutic efficacy of 4NSG; ii) conjugation of Gem to stearic acid may have imparted some degree of lipophilicity to 4NSG, which may have facilitated its delivery to cancer cells, and iii) encapsulation of 4NSG into SLN delivery system protected 4NSG and increase Gem payload.

## Conclusion

In this study, we demonstrated that 4NSG enhanced the therapeutic efficacy of Gem by improving its plasma metabolic stability and uptake into PCa cells. Chemical modification of Gem into 4NSG followed by formulation into 4NSG-SLN prolonged its systemic circulation and improved the bioavailability of Gem. Altogether, 4NSG-SLN showed a significant tumor inhibition in mice bearing Black and White patients’ pancreatic tumors compared with the GemHCl-treated mice bearing Black and White pancreatic tumors. Except for Black tumor sensitivity to 4NSG in the first week of treatment, no significant difference in 4NSG sensitivity was observed between Black and White tumors. Due to biological or genetic factors, an increased number of Black and White tumors may provide a definite difference in PCa status in our future studies.

## Supplementary Information

**Additional file 1: Supplementary Fig 1.** A (SF 1A). HPLC spectrum for detecting the purity of 4NSG at 98.8% with a wavelength of 254 nm. B (SF 1B). Nuclear magnetic resonance spectrum for 4NSG. (a) 4NSG proton nuclear magnetic resonance (1H NMR) peaks at 10.88 ppm (-CO-NH) and 1.38–1.04 ppm (-CH2)15 representing the amide linkage and long-chain methylene group contributed by stearic acid respectively. (b) amide carbonyl carbon in 4NSG displays a characteristic C-13 NMR peak at 174.55 ppm. C (SF 1C). Mass spectrum for 4NSG at 530.59 m/z.

**Additional file 2: Supplementary Fig 2.** (SF2 ) (a) Dynamic light scattering (DLS) graph showing the hydrodynamic particle size distribution of freshly prepared SLN with a mean diameter of 35 nm, (b) Dynamic light scattering (DLS) graph showing the hydrodynamic particle size distribution of freshlyprepared SLN with a mean diameter of 82 nm, (c) Graph of zeta potential distribution for 4NSG-SLN.

## Data Availability

The data that support the findings of this study are available on request from the corresponding author [EA].
