# Supplementary figures and images for: Biological evaluation of novel gemcitabine analog in patient-derived xenograft models of pancreatic cancer
Source: BMC Cancer. 2023 May 13;23:435. doi: 10.1186/s12885-023-10928-w (PMC10182601; doi:10.1186/s12885-023-10928-w)

**Supplementary Fig 1C (SF 1C)**


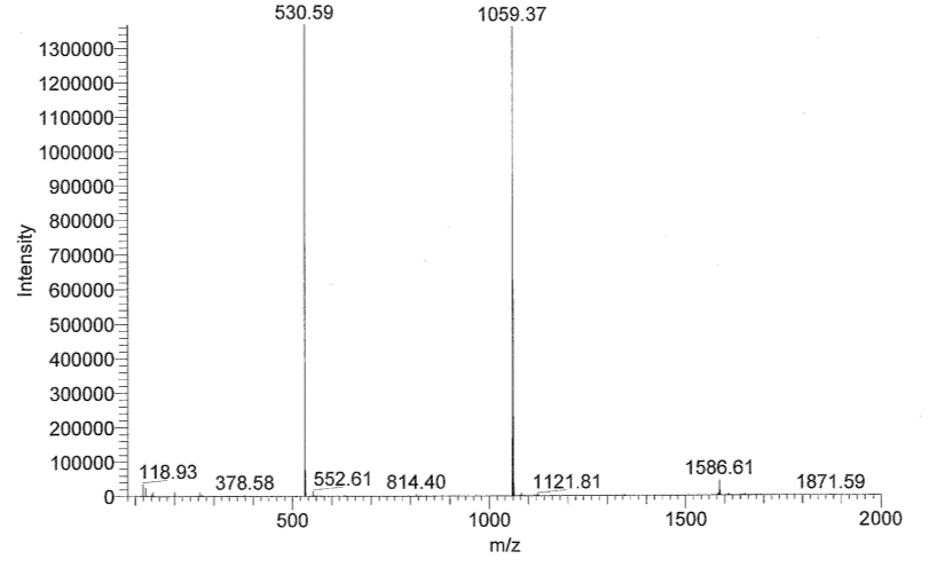


Legend

Mass spectrum for 4NSG at 530.59 m/z

Supplement: Supplementary file 1 — Additional file 1: Supplementary Fig 1. A (SF 1A). HPLC spectrum for detecting the purity of 4NSG at 98.8% with a wavelength of 254 nm. B (SF 1B). Nuclear magnetic resonance spectrum for 4NSG. (a) 4NSG proton nuclear magnetic resonance (1H NMR) peaks at 10.88 ppm (-CO-NH) and 1.38–1.04 ppm (-CH2)15 representing the amide linkage and long-chain methylene group contributed by stearic acid respectively. (b) amide carbonyl carbon in 4NSG displays a characteristic C-13 NMR peak at 174.55 ppm. C (SF 1C). Mass spectrum for 4NSG at 530.59 m/z. [file 12885_2023_10928_MOESM1_ESM.zip › Supplementary Fig 1C.docx]
