# Supplementary material for: Biological evaluation of novel gemcitabine analog in patient-derived xenograft models of pancreatic cancer
Source: BMC Cancer. 2023 May 13;23:435. doi: 10.1186/s12885-023-10928-w (PMC10182601; doi:10.1186/s12885-023-10928-w)
Supplement: Supplementary file 2 — Additional file 2: Supplementary Fig 2. (SF2 ) (a) Dynamic light scattering (DLS) graph showing the hydrodynamic particle size distribution of freshly prepared SLN with a mean diameter of 35 nm, (b) Dynamic light scattering (DLS) graph showing the hydrodynamic particle size distribution of freshlyprepared SLN with a mean diameter of 82 nm, (c) Graph of zeta potential distribution for 4NSG-SLN. [file 12885_2023_10928_MOESM2_ESM.docx]

**Supplementary Fig 2 (SF2 )**


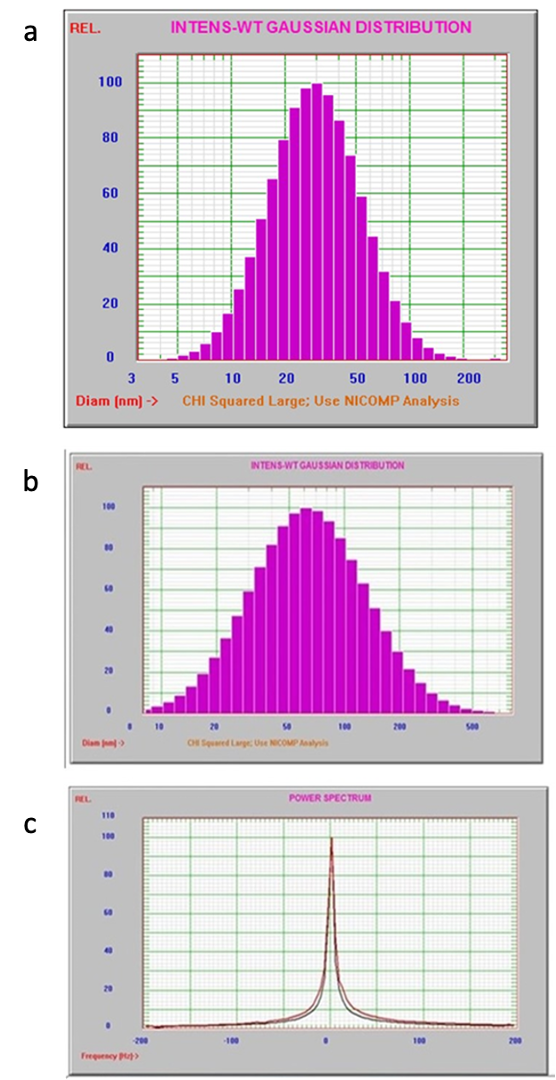


Legend

(a)Dynamic light scattering (DLS) graph showing the hydrodynamic particle size distribution of freshly prepared SLN with a mean diameter of 35 nm, (b) Dynamic light scattering (DLS) graph showing the hydrodynamic particle size distribution of freshly prepared SLN with a mean diameter of 82 nm, (c) Graph of zeta potential distribution for 4NSG-SLN
